# Tales of treatment and new perspectives for global health research on antimicrobial resistance

**DOI:** 10.1136/medhum-2020-011894

**Published:** 2020-09-18

**Authors:** Marco J Haenssgen, Nutcha Charoenboon, Patthanan Thavethanutthanawin, Kanokporn Wibunjak

**Affiliations:** 1 Global Sustainable Development, University of Warwick, Coventry, UK; 2 Institute of Advanced Study, University of Warwick, Coventry, West Midlands, UK; 3 Population Health Sciences, University of Bristol, Bristol, UK; 4 Mahidol Oxford Tropical Medicine Research Unit, Bangkok, Thailand

**Keywords:** exhibitions, health policy, science communication, social science, medical humanities

## Abstract

Global health champions modernism and biomedical knowledge but tends to neglect knowledge, beliefs and identities of rural communities in low-income and middle-income countries. The topic of antimicrobial resistance represents these common challenges, wherein the growing emphasis on public engagement offers a yet underdeveloped opportunity to generate perspectives and forms of knowledge that are not typically incorporated into research and policy. The medical humanities as an interdisciplinary approach to illness and health behaviour play a central role in cultivating this potential—in particular, through the field’s emphasis on phenomenological and intersubjective approaches to knowledge generation and its interest in dialogue between medicine, the humanities and the broader public.

We present a case study of public engagement that incorporates three medical humanities methods: participatory co-production, photographic storytelling and dialogue between researchers and the public. Situated in the context of northern Thailand, we explore subcases on co-production workshops with villagers, tales of treatment shared by traditional healers and dialogue surrounding artistic display in an international photo exhibition. Our starting assumption for the case study analysis was that co-produced local inputs can (and should) broaden the understanding of the sociocultural context of antimicrobial resistance.

Our case study illustrates the potential of medical humanities methods in public engagement to foreground cultural knowledge, personal experience and ‘lay’ sensemaking surrounding health systems and healing (including medicine use). Among others, the engagement activities enabled us to formulate and test locally grounded hypotheses, gain new insights into the social configuration of treatment seeking and reflect on the relationship between traditional healing and modern medicine in the context of antimicrobial resistance. We conclude that medical-humanities-informed forms of public engagement should become a standard component of global health research, but they require extensive evaluation to assess benefits and risks comprehensively.

## Introduction

Global health research and practice have been—and are increasingly—criticised for their reproduction of a hierarchy of knowledge that subordinates especially rural populations in low-income and middle-income countries (LMICs) to Western biomedical logic and to local medical elites.1, 2 For example, a report by Horton3 about a 2013 workshop on neocolonialism included positions that ‘western imposed (psychiatric) diagnoses, which ignore local understandings of distress, are “what imperialism is all about”’, while a recent *Nature* editorial commented that the inclusion of traditional Chinese medicine as a chapter in WHO’s International Classification of Diseases is ‘likely to backfire (and) risks legitimising an unfounded underlying philosophy’.4

Examples like these are reminiscent of critiques of modernism and neocolonialism in international development.5 More than 30 years ago, Arturo Escobar described that, ‘types of power and knowledge are being deployed (through Western disciplinary and normalising processes) in the Third World which try to insure the conformity of its peoples to a certain type of economic and cultural behaviour’.6 Considering the salience of health in the 2030 Sustainable Development Agenda, which aspires to be a more inclusive representation of global development than its predecessor the Millennium Development Goals,7 the persistence of a hierarchy between Western and biomedical knowledge on the one hand, and local and non-biomedical notions of health in LMICs on the other hand, would be problematic.

Antimicrobial resistance (AMR) is a global health topic that exemplifies this tension and the persistent hierarchy between ‘global’ (ie, Western biomedical) and ‘local’ knowledge. A top priority item on the global health agenda, the WHO Director-General has declared AMR (or ‘drug resistance’) as ‘one of the most urgent health threats of our time’.8 AMR involves the evolution of microbes like bacteria and viruses to withstand the medicine that humans use to treat them, thereby making them increasingly ‘drug resistant’ and the medicine less effective. This is in principle a naturally occurring process, but humans accelerate it by using antimicrobials (antibiotics, antivirals, antifungals, etc) in human and veterinary medicine, in agriculture, and through their leakage into the environment.

The global health response to AMR mirrors the biomedical interventionism with which postcolonial medicine has been characterised.9 Global policies to address AMR define it ‘as a global threat emerging from LMICs’10 and foreground individuals’ behaviour as one of the principal problems of a subject that connects humans, animals and the environment.11, 12 Remedial action focuses thus on awareness and education campaigns to change population behaviour especially in LMICs,13, 14 implying that knowledge and practices that deviate from a Western biomedical rationale—for instance, care from traditional healers during an illness—are problematic and require rectification.15–17

The global health response to AMR therefore continues to champion biomedical knowledge and to neglect or otherwise subordinate the knowledge, beliefs and identities of rural communities in LMICs. At the same time, the growing emphasis on ‘public engagement’ (a form of ‘patient and public involvement’) among health researchers and medical research funders has in principle the potential to break down or at least undermine hierarchical relationships between medical elites and local populations.18–22 However, rather than establishing dialogue and challenging this hierarchy, public engagement activities have thus far primarily been instrumentalised to impose global health agendas on local populations (eg, through theatre plays23; see ‘Background’ section for further explanation).

This need not be the case. The medical humanities intersect with the popular practice of public engagement, emphasising in particular creative co-production research that uses methods including theatre, storytelling or artistic production (we related to the medical humanities in this paper especially through creative, participatory and dialogical approaches from within the visual arts and social sciences/cultural studies branches of the medical humanities).24, 25 Medical humanities scholarship thereby shares an interest in the role of experience and expression of illness and healing,26–29 and it has an important role to play in broadening debates away from reductionist towards a holistic and context-sensitive understanding of complex medical phenomena like AMR.30–32 At the same time, explorations building on artistic and creative processes do not exist in opposition to but rather enable dialogue with established medical notions and conventions, giving them new perspectives and interpretations.33–36

Building on this logic, this paper presents a case study of public engagement under the umbrella of the medical humanities in which we examine new and locally grounded perspectives on the sociocultural context of AMR and its related topics of medicine use and health systems. Our research question is, ‘*Can medical humanities approaches challenge hierarchies and promote engagement in global health research on antimicrobial resistance?*’ To answer this question, we employed a case study design to illustrate the diverse ways in which public engagement can reveal perspectives and forms of knowledge that are not typically incorporated into AMR research and policy—in particular cultural knowledge, personal experience and ‘lay’ sensemaking surrounding health systems and healing (including medicine use).37–40 We focus on these aspects of knowledge as they are typically replaced by biomedical assumptions in AMR policies, whereby our case relates in particular to the sociocultural context of human antibiotic use (a major determinant of AMR).

We consider in the context of northern Thailand co-production workshops, storytelling and dialogue surrounding artistic display as vehicles for a bottom-up process for knowledge generation under the umbrella of participatory (rather than instrumental) engagement.41 The objective of this paper is thereby not to influence global and local AMR policy directly, but to offer a case that demonstrates how the inclusion of medical humanities methods in global health research (via public engagement) can open up new and locally grounded perspectives for thinking about the complex issue of AMR and its related and seemingly established topics of medicine use and health systems. We thereby contribute to a small but growing body of medical humanities research that relates directly to AMR and the broader interface of humans and health systems.42–47

## Background

Research on population health behaviour in AMR mobilises conventional public health research methods. We review these methods in this section and argue that they risk reproducing a hierarchical relationship which subordinates local medical knowledge and traditional forms of healing in LMICs to the biomedical model of health that is prominent in high-income countries and among local medical elites—which underscores the important role of the medical humanities in this space.48, 49

Standard forms of AMR knowledge generation with particular relevance to the current case are public awareness surveys and knowledge, attitude and practice (KAP) surveys. For example, one of the most influential documents in the context of awareness-related global AMR policy is the WHO’s *Antibiotic resistance: multi-country public awareness survey*.50, 51 Based on online and face-to-face surveys in 12 countries and using a range of knowledge-testing questions, the survey argues that, ‘it is critical that people understand the problem (of drug resistance), and the way in which they can change their behaviour’.52 KAP surveys are similarly prominent in the field of public health AMR research,53 including (with a focus on antibiotics) contexts as diverse as the studies by Belongia *et al* 54 on patients’ antibiotic use for respiratory illnesses in the USA, by Yu *et al* 55 on parental antibiotic use for their children in China, or by Awad and Aboud56 on the general public’s antibiotic use in Kuwait.

Public awareness and KAP surveys as mainstream tools for global health knowledge generation typically conclude that awareness needs to be raised, and call on individuals’ responsibility to change antimicrobial-related health behaviours.57–61 A major problem of these approaches is that problematic antimicrobial use is framed in terms of knowledge and attitudes, and the notions of what constitutes ‘desirable knowledge’ are typically imposed by health researchers who implicitly assert superiority of modern over local and traditional forms of knowledge.62 Yet, such studies devote little if any concern towards the social and ethical antecedents of current behaviour (and the corresponding consequences of intervention) in LMICs—for instance, the historical role of drug promotion, the precarious balance between antimicrobial ‘access and excess’ or culturally specific notions as to what constitutes ‘good care’.63–65

In contrast, recent social sciences and interdisciplinary research on AMR has pointed out non-individual components of antimicrobial use that reflect on the broader sociocultural context of AMR. For example, Chandler66 describes the interconnectedness of AMR across the domains of human, animal and environmental health and the social role of antimicrobials as ‘infrastructure’ that contributes to the functioning of market economies; Hinchliffe *et al* 67 indicate how Bangladeshi shrimp and prawn farmers adapt their antimicrobial use in response to economic uncertainty and perceived disease risks and Chuengsatiansup and Limsawart68 analyse the tensions between administratively defined borders and their history, enactment and continued negotiation in the control of drug-resistant tuberculosis in the border area of Thailand and Myanmar. However, the global health discourse around AMR has not yet been infused with these perspectives and continues to portray a dominance of Western high-income countries’ priorities and solutions, LMICs as source of a global problem and individuals’ knowledge and behaviour as critical targets for intervention.69, 70

Alternative forms of knowledge generation could broaden the global health discourse around AMR. As the aforementioned examples illustrate, exploratory qualitative research or participatory research methods could in principle offer insights into the sociocultural context of AMR and thereby provide an avenue to challenge the mainstream framing of AMR and the implied hierarchy of medical knowledge and practice. However, qualitative research in public health often remains limited to examining people’s attitudes and knowledge akin to quantitative public awareness surveys.71–73 Similarly, ‘participatory methods’ or ‘public engagement’ in public health research are typically instrumental means with an emphasis on health education provision, on ‘mobilising’ communities to change their health behaviour, and/or on building trust and legitimacy of health research locally.74–81

Global health scholars have argued that qualitative research and public engagement involving the co-production of knowledge with inputs from target populations can broaden understanding and open new directions for debate.82, 83 Yet, in public health and global health research, these methods have a tendency to retain biomedical assumptions, to fall short of their potential to challenge hierarchies of knowledge and even reproduce neocolonial relationships in global health.84, 85

This persistent challenge underlines the important role of the medical humanities.86, 87 Aside from historical analysis or literary interpretations,88, 89 common methods in the medical humanities have included narration, artistic means such as theatre and photography and also directed qualitative approaches like focus group discussions to interrogate the nature of illness, healing and people’s relationship to health systems.90–95 Unlike common public health research methods and instrumental forms of public engagement, a medical humanities approach thereby helps reveal subjective truths that are often overlooked in biomedical perspectives, and thus challenge hierarchies of knowledge between clinical ‘experts’ and non-medical ‘lay’ people.96–99 For example, Cole and Gallagher100 argue in the context of clinical neuroscience that the medical humanities, in the form of first-person narratives, ‘can complement the clinical third-person approach, and in some cases lead to better understanding and point towards further empirical work itself’. Our focus on the medical humanities in the present study thereby builds on the understanding that the joint consideration of medicine, participatory research and artistic forms of expression enables us to generate new and critical knowledge about complex global health phenomena like AMR.101–103

## Materials and methods

### Study design

We employed an embedded case study research design, in which we focused on three public engagement components of an interdisciplinary research project on rural health behaviours and drug resistance in northern Thailand and southern Lao PDR (each component is a ‘subcase’ embedded within the larger public engagement case study).104, 105 Case studies in the medical humanities help illustrate conceptual and methodological applications, present close-up experiences of (often otherwise neglected or marginalised) research participants and open up new perspectives on medical topics.106–111 These designs also often combine different methodological approaches within the same analysis (eg, Hume *et al*,112 who combine insights from historical, ethnographic and creative research).

In relation to the research question, our starting assumption for the case study analysis is that co-produced local inputs can (and should) broaden the understanding of the sociocultural context of AMR. We included the three subcases to illustrate the diverse ways in which public engagement can reveal perspectives and forms of knowledge that are not typically incorporated into AMR research and policy—in particular cultural knowledge, personal experience and ‘lay’ sensemaking.113–116

We situate the case study research design firmly within the medical humanities. Aside from using medical humanities methods of knowledge generation (see section ‘Data collection and analysis’ for further explanation), our public engagement activities operationalised the phenomenological and intersubjective approach of the medical humanities through qualitative and participatory techniques that treat participants as expert informants and, where possible, let them choose the terms of the conversation.117, 118 At the same time, the case study considers the dialogue with the medical profession by relating qualitative interpretations to quantitative patterns of health behaviour and to the global health discourse around AMR, and by considering the costs and risk of incorporating our approach into public engagement practice.119 Our mixed-method approach is thereby compatible with both the case study research design and its application in the medical humanities.120

Several recent examples have adopted approaches similar to our current study: for instance, Macnaughton and Carel121 use case examples to describe how a critical medical humanities approach can help understand the phenomenon of breathlessness beyond its clinical dimensions—based, among others, on reflections from collaborative meetings between medical researchers, artists and social scientists. Barbieri *et al* 122 present a qualitative case study of patients in Italian paediatric wards, whereby the production and interpretation of semifictional autobiographic narratives offered child patients a channel to communicate to clinicians their personal experience of illness and healthcare needs. A case of HIV-related public engagement in South Africa by Treffry-Goatley *et al* 123 further uses a combination of qualitative (focus group discussions, observation) and quantitative methods (surveys) to understand the role of digital storytelling in promoting health literacy. We therefore build on an established body of work in the medical humanities.

### Case overview

The public engagement components in this project involved workshops in three villages and the collection and exhibition of photographic stories of healing in Chiang Rai province in northern Thailand.

The workshops took place in three villages in Chiang Rai province, namely Chiang Rai village, Chiang Khong village and Mae Fah Luang village (pseudonyms named after the districts in which they were located). The two main objectives of the workshops were, first, to share with villagers some ideas and concepts about antibiotics and drug resistance (based on material from the WHO124), without assuming that their current knowledge and behaviours were in any way deficient; and, second, to enable our research team to learn from the villagers about the local context of medicine and healing and how the antibiotic-related information has been received. The half-day workshops involved 20–35 adults per village, who were recruited in a combination of purposive and snowball sampling to ensure spatial and ethnic diversity of the workshop participants (however, all of the attendees had Thai language abilities, which limited the representativeness of the workshops).125 The workshop activities involved, in chronological order:

1. an ice-breaking activity to create an open and positive atmosphere;
2. a community map drawing activity to represent different types of healthcare providers;
3. a pile sorting activity to understand conceptions and categories of medicines;
4. a drug resistance-themed chair game to illustrate the evolution of bacteria;
5. a traditional pop song with adapted lyrics to illustrate WHO messages to seek advice from medical practitioners;
6. a role-playing activity to illustrate the relationship between antibiotic use and drug resistance;
7. a poster-making activity as a feedback mechanism and to understanding participants’ interpretations of the workshop content (see Charoenboon *et al* 126 for a detailed description of the workshops).

**Figure 1:**
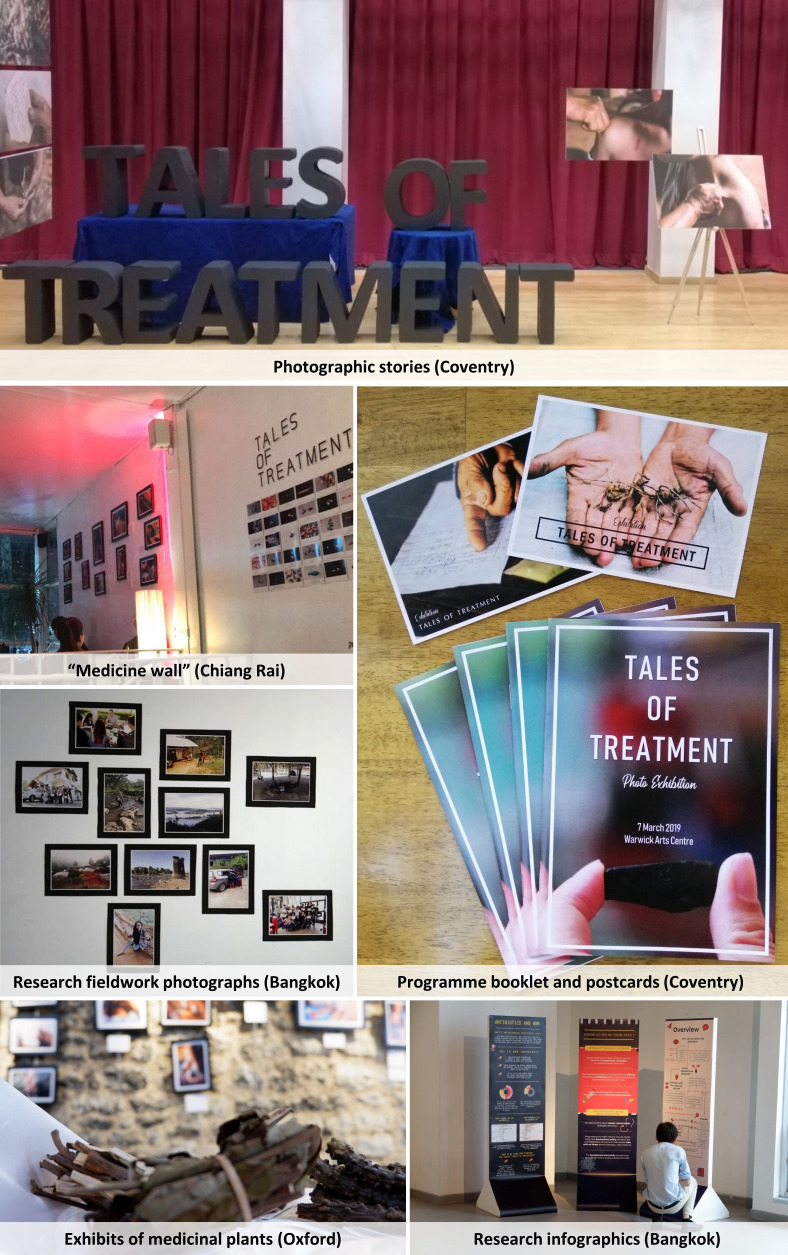
Impressions of ‘Tales of Treatment’ exhibition elements. Source: Authors.

The workshops took place alongside larger health behaviour surveys in Chiang Rai. Feedback from our field research team also revealed that our survey questionnaire did not capture important aspects of local healing. As the project surveyed 72 villages in Chiang Rai, the team shared experiences of herbalists curing broken bones and spiritual healers summoning ghosts. What was the meaning and significance of these practices, and what would the survey category ‘traditional healer’ mean for villagers? To investigate these questions further, the research team revisited some of the villages to document local stories of healing from the perspective of traditional healers (with their permission). The resulting narratives were exhibited in the ‘Tales of Treatment’ photo exhibition series in Bangkok (Art Gallery g23), Chiang Rai (Tai tea shop and bar), Oxford (Green Templeton College) and Coventry (Warwick Arts Centre) between July 2018 and March 2019 (note that the international exhibition was not intended to ‘validate’ local knowledge through urban elites but to encourage dialogue and reflection about what healing is on an international scale with diverse audiences).127 The content of the exhibitions varied slightly by location (considering available space and logistics; see figure 1 for illustrations) and included:

- Fifteen photographic stories with Thai/English captions and guided tours by the research team (all four exhibition sites).
- Exhibits of pharmaceuticals and medicinal plants (Bangkok, Chiang Rai, Oxford).
- ‘Medicine wall’ of pharmaceutical images and local notions of medicines (Bangkok, Chiang Rai, Oxford).
- Programme booklets and souvenir postcards (Coventry).
- Research fieldwork photographs (Bangkok, Oxford).
- Research infographics, word clouds and/or animated presentations (Bangkok, Chiang Rai, Oxford).

### Data collection and analysis

We used primary qualitative and quantitative data to document and explore how knowledge co-production challenged our own expectations as survey researchers and contributed to new perspectives on the sociocultural context of AMR. We will present our research according to subcases representing the three medical humanities methods contained in this case study: participatory co-production, photographic storytelling and dialogue between researchers and the public. The groups with whom we engaged included Chiang Rai villagers, traditional healers in Chiang Rai and urban audiences of the photo exhibitions in Thailand and the UK. The data sources and involved groups are described in the remainder of this section, and a summary is provided in table 1.128

**Table 1:** Data overview

Subcase no.	Method	Involved groups	Sample
1	Co-production	Rural population in Chiang Rai	20–35 workshop participants per village
			2422 survey respondents (1264 in census survey, 1158 in provincial survey)
2	Storytelling	Traditional healers in Chiang Rai	15 photographic narratives
3	Dialogue	International exhibition attendees	500+ across Chiang Rai, Bangkok, Oxford and Coventry 23 feedback form respondents at Warwick Arts Centre

In the first subcase, we collected observational data to document the inputs from workshop participants and to formulate locally grounded hypotheses about medicine use (documented in digitised fieldnotes and audio-visual material including photographs and video). To illustrate the applicability of the co-produced knowledge for understanding the sociocultural context of AMR, we tested these hypotheses using rural health behaviour survey data from the main research project (for details on sampling strategy, please refer to the published study protocol referenced here129; survey data collection was through electronic questionnaires on tablet computers in face-to-face survey interviews). In brief, the rural health behaviour survey data used in this analysis had two components, namely:

- Two rounds of complete census surveys in the three workshop villages. As complete census surveys, the entire adult population of the three villages was invited to participate in both survey rounds (total adult population est.: 694), with an average response rate of 91%. The workshops took place in between the two survey rounds but were not intended to be an ‘intervention’—rather, they were a public engagement activity and were therefore not implemented as a (quasi-)experiment.
- A provincial-level representative rural survey. This survey used a three-stage stratified random sampling design to represent the rural adult population of Chiang Rai province (522 000 adults according to census data).130 We selected five districts purposively (stage 0; selected for diversity within the province), within each of which we selected six primary sampling units randomly (stage 1; stratified by distance to nearest town), followed by the random selection of at least 30 households per primary sampling unit (stage 2; interval sample and stratified by village segments), and by the simple random selection of one per every five household members (stage 3). The total sample comprised 1158 adults.

As shown in the questionnaire (see online supplemental material), the surveys collected data on the individual level (eg, demographic attributes, antibiotic knowledge and attitudes), illness level (eg, healthcare choices) and the step level (eg, medicine use at each step of the treatment-seeking process). We tested the hypotheses using descriptive statistical analysis, comparing responses across groups and, where appropriate, performing Pearson’s χ^2^ tests whether these differences were statistically significant. We first applied the hypotheses to the village(s) where they arose, then to all three workshop villages (using the first or both survey rounds depending on whether data analysis took place on the individual or illness level)131 and subsequently to the representative sample of rural Chiang Rai province.

10.1136/medhum-2020-011894.supp1 Supplementary data

For the second subcase, the storytelling component involved stories from traditional healers in northern Thailand, whereby we considered photography and oral accounts as interlinked elements of a narrative in line with McKechnie.132 The emphasis on these stories as ‘tales’ implies that the activity was not aimed to uncover an objective reality but rather a systems of experiences, relationships and subjective truths that can help challenge entrenched and simplistic outsiders’ perspectives on such topics as health systems, medicine use and behaviours linked to AMR.133, 134 Health systems thereby refer to pluralistic landscapes of in-/formal healthcare providers (including public and private doctors, unregulated medicine sellers, traditional healers or carers within a family network),135, 136 which global health researchers and policy makers in AMR often simplify as organised and formal healthcare provision.137, 138

The narratives were captured by a Thai photographer (PT) and a chronicler (KW), who were both members of the research survey team (ie, data were collected via audio-visual material and digitised written narratives). Based on the experiences of the survey team, we revisited 15 traditional healers and received their permission to document their tales through written narratives and audio-visual records of the people, artefacts, process and context of traditional healing in any way the healers wished to present these aspects (yielding 61 GB of visual material). The exhibition curator and survey team manager (NC) translated the narratives into English and edited them for consistency. Out of the 15 narratives (which can be accessed at https://tinyurl.com/talesoftreatment), we reflected in this subcase on three that related directly to AMR, medicine use and health systems.

The photographic narratives were presented at the ‘Tales of Treatment’ exhibition. Understanding that display plays an integral role in the dialogue between art and public audiences,139 we drew in this third subcase on verbal and written feedback from the photo exhibitions and reflected as a team on audience reactions and the potential impact of the public engagement activity (quantitative feedback was digitised in a spreadsheet, written feedback was digitised via word processing software or as photographs of guest book entries).140

In summary, the study team reviewed and analysed descriptively the qualitative and quantitative material against the objective to illustrate the diverse ways in which public engagement can reveal perspectives and forms of knowledge that are not typically incorporated into AMR research and policy. The primary analytical focus was on how co-produced local inputs could broaden the understanding of the social context of AMR (ie, constant critical comparison with the global health AMR discourse), which was directed by the principal investigator and supported by all coauthors (local Thai social researchers). Note that none of the data collection and analysis methods presented here constitutes a formal evaluation of the public engagement activities. However, based on experiences in the medical humanities literature and our previous public engagement projects, we were conscious that unstructured interactions with narratives and artistic products could entail unintended and potentially adverse interpretations and behavioural responses, which we explicitly incorporated as analytical angles as well.141–143

### Patient and public involvement statement

This publication reports a case of public involvement for informing global health research. The broader project was further based on preceding qualitative research with local northern Thai patients and the general public, which revealed the need for more grounded sociomedical studies that respond to participants’ viewpoints and cultural context. Members of the Thai public were involved in this project through cognitive interviewing to inform the survey design and the interpretation of the data, Thai participants were involved in local workshops during the study period to improve our understanding of local medicine uses and health behaviours, Thai villagers shared their stories about traditional healing on their own terms to broaden the scope of the standardised survey and Thai and UK publics were involved in the photo exhibitions where they were able to relay feedback and their interpretations of the photo stories. Note that this study did not specifically focus on patients but on members of the general public.

## Results

We report separately on the knowledge co-production workshops and storytelling activities, using observations from co-production and engagement activities, primary survey data and event feedback. The results illustrate how insights and reflections sparked by the direct input from research populations and through the engagement of the public can broaden debates and viewpoints within the field of global health. However, the results also hint at the limitations and potential risks of a co-production approach, which we address separately in the subsequent ‘Discussion’ section.

### Subcase 1 (co-production): participatory workshops

Using the example of the medicine pile sorting activity, we exemplify in this section how interactions between the research team and the workshop participants helped shape our understanding of medicine use in rural Chiang Rai. The pile sorting session was one of three workshop activities in which the participants were directly asked to educate the research team, and it lent itself for this illustration due to its focus on the meaning and uses of medicine in the context of AMR (the mapping activity focused on the healthcare landscape, and the poster making activity focused on participants’ interpretations of the workshop content). The sorting activity thereby enabled at least three hypotheses about the relationships between the local social context, notions of medicine and treatment-seeking behaviour—which we outline and test below (for details on local notions of ‘antibiotics’, see a report of the main research project referenced here).144

#### Theme 1: ‘antibiotics that you can buy’

Our first example involved workshop participants in Mae Fah Luang village. The participants described how they categorise different types of antibiotics into the groups “you can buy this medicine over the counter” and “you need a prescription from a doctor to obtain this medicine”. These categories related directly to global health awareness campaigns, as for instance, WHO advocates that antibiotics should only be used ‘when prescribed by a certified health professional’.145 Based on the input from the villagers, we therefore hypothesised that,

H1: Villagers’ attitudes towards buying antibiotics over the counter differ depending on the types of antibiotics that they recognise.

Our survey data contained the names with which people referred to antibiotics.146 While the survey did not ask respondents to classify antibiotics into the categories of ‘can buy’ and ‘cannot buy’, we could at least learn from this data whether they were familiar with common colloquial names for antibiotics as ‘anti-inflammatory medicine’ (‘ยาแก้อักเสบ’ or ‘*yah kae ak seb*’). The survey further asked knowledge and attitude questions corresponding to antibiotic awareness-raising material from the WHO, including whether there are situations in which the respondent would buy antibiotics over the counter—‘desirable’ responses being those that fell in line with the WHO position (*not* to judge whether their behaviour was inappropriate or unjustified), meaning that the respondent would not buy this medicine without a prescription.147 If the hypothesis holds, then we would expect to see different attitudes to over-the-counter antibiotic purchases depending on how the respondents referred to the medicine.

Table 2 shows the most commonly mentioned names of the three antibiotic images presented to the survey respondents (all respondents were shown the same images). The colloquial name ‘anti-inflammatory’ dominated the local notions of antibiotics. Owing to the ethnic diversity of Mae Fah Luang village, several other local language descriptions unbeknownst to us circulated alongside notions like ‘germ killer’, capsule medicine, cough medicine, pain reliever or vernacularized generic antibiotic names like ‘*amoxi*’ (for amoxicillin) and ‘*colem*’ (for chloramphenicol). The column ‘“desirable” attitude’ indicates whether people’s attitude aligned with WHO positions, depending on how the respondents described the medicine presented to them. Because the respondents could mention several different names, and because the mentioned names were likely correlated with respondents’ personal characteristics (eg, ethnic background, language ability, education), these data should be interpreted with caution. However, a pattern emerged in which the technical term ‘antibiotic’ was associated with a relatively high share of ‘desirable’ attitudes. Consistent with hypothesis (H)1, different notions were linked systematically to very different attitudes, for instance, ‘*colem*’ was linked to levels of ‘desirability’ ranging from 26.7% (all workshop villages) to 42.4% (provincial survey), whereas ‘*amoxi*’ ranged from 65.4% (provincial survey, not shown) to 83.3% (all workshop villages). Attitudes across the top-10 mentions ranged from 33.3% to 100.0% ‘desirable’ (Mae Fah Luang), from 26.7% to 83.3% (all workshop villages) and from 23.7% to 73.5% (provincial survey).

**Table 2:** Top 10 responses to describe pictures of common antibiotics used in Chiang Rai province, and the corresponding share of respondents that would refrain from buying the medicine over the counter (‘desirable’ attitude)

Rank	Mae Fah Luang village (first survey round; n=155)			All three workshop villages (first survey round; n=497)			Rural Chiang Rai province (n=1098)		
	Name	Mentioned	‘Desirable’ attitude	Name	Mentioned	‘Desirable’ attitude	Name	Mentioned	‘Desirable’ attitude
1	Anti-inflammatory	70.3%	48.6%	Anti-inflammatory	72.4%	53.6%	Anti-inflammatory	86.4%	55.0%
2	Other (unknown) names	25.8%	70.0%	Other (unknown) names	26.8%	53.4%	Do not know the name of this medicine	10.3%	73.5%
3	Do not know the name of this medicine	14.8%	65.2%	Do not know the name of this medicine	12.7%	65.1%	Germ killer	10.3%	55.0%
4	Germ killer	7.1%	72.7%	Germ killer	5.0%	72.0%	Antibiotics	7.0%	67.9%
5	Capsules/Medicine in general	5.8%	77.8%	Capsules/Medicine in general	3.8%	52.6%	Heromycin, TC-Mycin, etc.	5.6%	39.4%
6	Amoxi (amoxicillin)	3.2%	80.0%	Colem (chloramphenicol)	3.0%	26.7%	Colem (chloramphenicol)	4.8%	42.4%
7	Cough medicine	1.9%	33.3%	Pain reliever	2.4%	58.3%	Capsules/Medicine in general	4.6%	46.4%
8	Pain reliever	1.9%	66.7%	Antibiotics	2.2%	81.8%	Colour reference	3.1%	27.3%
9	Colem (chloramphenicol)	1.9%	33.3%	Amoxi (amoxicillin)	1.2%	83.3%	Pain reliever	2.5%	52.3%
10	Antibiotics	1.3%	100.0%	Cough medicine	1.0%	40.0%	Other non-antibiotic medicine	1.7%	24.8%

See questionnaire in online supplementary material for pictures of common antibiotics. Only including respondents who recognised the medicine shown. Multiple mentions per respondent possible. Provincial-level results are population weighted using census data.

Source: Authors; derived from survey data.

Although the patterns were indicative rather than conclusive, the data provided circumstantial evidence in support of H1, namely that different names given to antibiotics were linked to different attitudes about antibiotic purchases. Future research could incorporate this aspect more systematically to understand which antibiotics villagers may be more inclined to procure over the counter—regardless of whether they have a biomedical understanding of antibiotic medicine.

#### Theme 2: ‘prescription medicine for children’

Our second example relates to another insight from the pile sorting activity that we encountered in Mae Fah Luang village and Chiang Rai village. We learnt that villagers categorised medicine into ‘medicine for adults’ and ‘medicine for children’. According to the workshop contributions, people would be extra careful with ‘medicine for children’ (follow instructions closely, and indeed only receive it against prescription), whereas the workshop participants would buy ‘medicine for adults’ for themselves over the counter. Antibiotics fell into both categories, which led us to hypothesise that,

H2a: If children receive antibiotics, these antibiotics are more likely to originate from formal healthcare providers.

H2b: If children receive antibiotics, these antibiotics are more likely to be used in accordance with their instructions.

The surveys elicited healthcare pathways during an acute illness or accident within the 2 months prior to the survey interview—both for the respondents and for children under their supervision (children were defined in the survey as anyone below 18 years of age; adults would thereby report the illness episodes of ‘children’ under their supervision). At each step of the process, the respondent could indicate whether any medicine was received, whether it was taken in line with the instructions received, and whether the medicine was finished.148 To operationalise these data for the hypothesis, we considered (a) illness episodes involving at least one dose of antibiotics, (b) whether these antibiotics originated from formal (public or private clinics/hospitals/pharmacies) or informal sources (unregulated healthcare providers including, grocery stores, traditional healers), (c) whether at least one set of antibiotics remained unfinished and (d) whether respondents took at least one set of antibiotics as recommended. We examined these factors initially for the two workshop villages where these statements originated (focusing on the first survey round prior to the workshop), and then expanded the analysis to the full sample of illness episodes in both the workshop villages and the provincial survey. To test whether these differences were statistically significant, we performed Pearson’s χ^2^ tests.

Table 3 reports that adults consumed antibiotics in 12.2%–19.2% of all recorded illness episodes, whereas children’s antibiotic consumption ranged from 13.2% to 24.5%. Within these episodes, the sources of children’s antibiotics were systematically more likely to include formal healthcare providers, whereas adults were more likely to use antibiotics from informal sources. The Pearson’s χ^2^ tests indicated that use of antibiotics from formal sources was statistically significantly different between adults and children at least at the 10% level (Mae Fah Luang and Chiang Rai: p=0.070; all workshop villages: p=0.041, provincial level: p=0.083). The difference in informal antibiotic use, too, was statistically significant, except in the provincial data (Mae Fah Luang and Chiang Rai: p=0.070; all workshop villages: p=0.088, provincial level: p=0.235). In contrast, none of the differences in completing antibiotic courses or adhering to instructions was statistically significant for any of the three samples.

**Table 3:** Comparison of adults’ and children’s antibiotic sources and use during acute illnesses and accidents

	Mae Fah Luang and Chiang Rai villages (first survey round)			All three workshop villages			Rural Chiang Rai province		
	Adult	Child	P value	Adult	Child	P value	Adult	Child	P value
	All illness episodes								
Number	229	68		697	168		696	156	
% received antibiotics	12.2%	13.2%	0.825	14.3%	16.7%	0.447	19.2%	24.5%	0.321
	All antibiotic use episodes								
Number	28	9		100	28		125	31	
% of antibiotic use episodes received from formal sources	71.4%	100.0%	0.070	75.0%	92.9%	0.041	83.6%	100.0%	0.083
% of antibiotic use episodes received from informal sources	28.6%	0.0%	0.070	26.0%	10.7%	0.088	18.3%	6.1%	0.235
% of illness episodes with at least one instance of unfinished antibiotics	42.9%	44.4%	0.933	40.0%	39.3%	0.946	36.5%	48.6%	0.338
% of episodes with at least one instance of strict adherence to antibiotic instructions	64.3%	77.8%	0.452	67.0%	67.9%	0.932	72.2%	70.8%	0.908

Data on illness episode level. Multiple illness episodes per respondent possible. Provincial-level results are population weighted using census data. P values calculated using Pearson’s χ^2^ test. ‘Child’ refers to illness episodes of anyone below 18 years of age; adults would report the illness episodes of ‘children’ under their supervision.

Source, Authors; derived from survey data.

However tentative, these findings help broaden the understanding of antibiotic use (and potentially the identification of priority or high-risk target groups) in different segments of the population. Children were indeed more likely than adults to receive antibiotics from a formal healthcare provider, which is consistent with H2a. In contrast, the limited survey evidence did not indicate that the distinction between ‘medicine for adults’ and ‘medicine for children’ translated into stricter adherence to antibiotic use instructions (H2b), but it is noteworthy that adult illness episodes (n=125) across Chiang Rai province had a more than 10-percentage-point lower rate than children (n=31) in terms of non-completion of an antibiotic course (36.5% vs 48.6%).

#### Theme 3: ‘assertive youth’

Our last example again relates to demographic differences in medicine use. In Chiang Rai village, workshop participants reported that young adults would more commonly engage in arguments and assert their position vis-à-vis figures of authority, like doctors or elders. This may be generic judgement of older towards younger generations,149 but we could argue that older people—when they were younger—had experienced a different health system and different social hierarchies than today’s youth. This raised the question whether age gradients may reflect different patient-health system relationships across generations, and with them different patterns of antibiotic use. We therefore hypothesised that,

H3a: Younger adults are more likely to source antibiotics from informal healthcare providers.

H3b: Younger adults are less likely to use antibiotics in accordance with their instructions.

To test these hypotheses, we again examined first the initial survey round from Chiang Rai village, followed by the complete workshop village sample and provincial survey. We used the same analysis categories as in the previous section (a: illness episodes involving at least one dose of antibiotics, b: antibiotics from formal/informal sources, c: unfinished antibiotic courses, d: adherence to antibiotic instructions) and analysed the differences across five age groups, namely 18–24, 25–34, 35–44, 45–59 and 60+ years. We used Pearson’s χ^2^ tests for differences across age groups.

**Figure 2:**
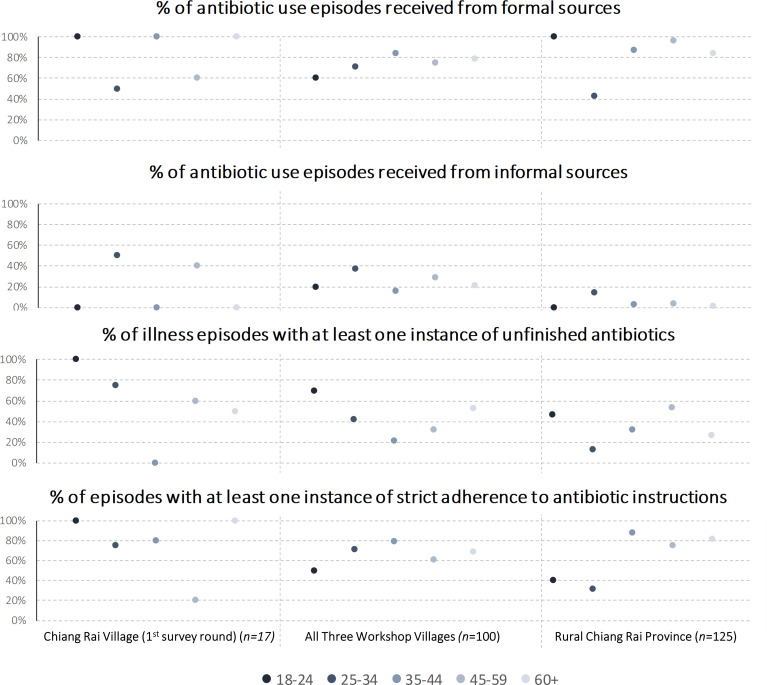
Comparison of antibiotic sources and use during acute illnesses and accidents across five age groups. Source: Authors; derived from survey data. Data on illness episode level. Multiple illness episodes per respondent possible. Provincial-level results are population weighted using census data.

Figure 2 presents the results of the group comparison (see online supplementary appendix table A1 for detailed results including Pearson’s χ^2^ tests). Because of the small sample of 17 illness episodes in Chiang Rai village, we focused the analysis on the larger workshop village sample (100 episodes) and the provincial survey (125 episodes). Among the workshop villages, the age group 35–44 years exhibited the highest degree of formal antibiotic use (84.2%; sample average: 75.0%) coupled with the lowest incidence of informal antibiotic consumption (15.8%; sample average: 26.0%), the lowest incidence of leaving antibiotics unfinished (21.1%; sample average: 40.0%) and the highest rate of instruction adherence (78.9%; sample average: 67.0%). Both younger and older age groups indicated higher informal use and less strict adherence to antibiotic regimes. However, only the difference in unfinished antibiotic courses was statistically significant at p=0.020. While the provincial-level age group differences were statistically significant in the cases of formal antibiotic use (p=0.001) and instruction adherence (p=0.006) (informal antibiotic use: p=0.418; unfinished antibiotics: p=0.110), the patterns across age groups were distinctly different from the workshop village sample: the age group 25–35 years had notably below-average formal antibiotic use (42.4%; sample average: 83.6%), above-average informal antibiotic use (14.2%; sample average: 4.6%) and below-average unfinished antibiotics (13.1%; sample average: 36.5%) and adherence to instructions (31.3%; sample average: 72.2%). The younger age group of 18–24 years did not follow this trend and mostly corresponded to the remainder of the sample.

10.1136/medhum-2020-011894.supp2 Supplementary data

The mixed patterns across the samples suggest caution in supporting or rejecting the hypothesis, but the data did suggest that antibiotic use behaviour was likely to have an age dimension. Further qualitative research would allow us to investigate whether these patterns are systematic, and, if so, if they related to different age groups’ assertiveness (eg, driven by formal education) as argued in the workshops, or whether they were a result of different meaning of medicines across generations. In summary, the three workshop themes presented here illustrate how the public engagement activity helped co-produce locally grounded hypotheses relating to medicine use as a specific aspect of the sociocultural context of AMR.

### Subcase 2 (storytelling): of healing and treatment in northern Thailand

In this second subcase, we exemplify how storytelling techniques as part of public engagement helped shed light on the broader sociocultural context of AMR. The stories narrated in the ‘Tales of Treatment’ exhibition illustrate insights about local healing, health systems and reflections on global health that would not otherwise have emerged from the broader research project. The narratives did not intend to present superior or effective forms of treatment but rather to document disappearing perspectives and practices of healing in Chiang Rai, following the bottom-up process of participatory engagement in which traditional healers chose the focus of their stories.

**Figure 3:**
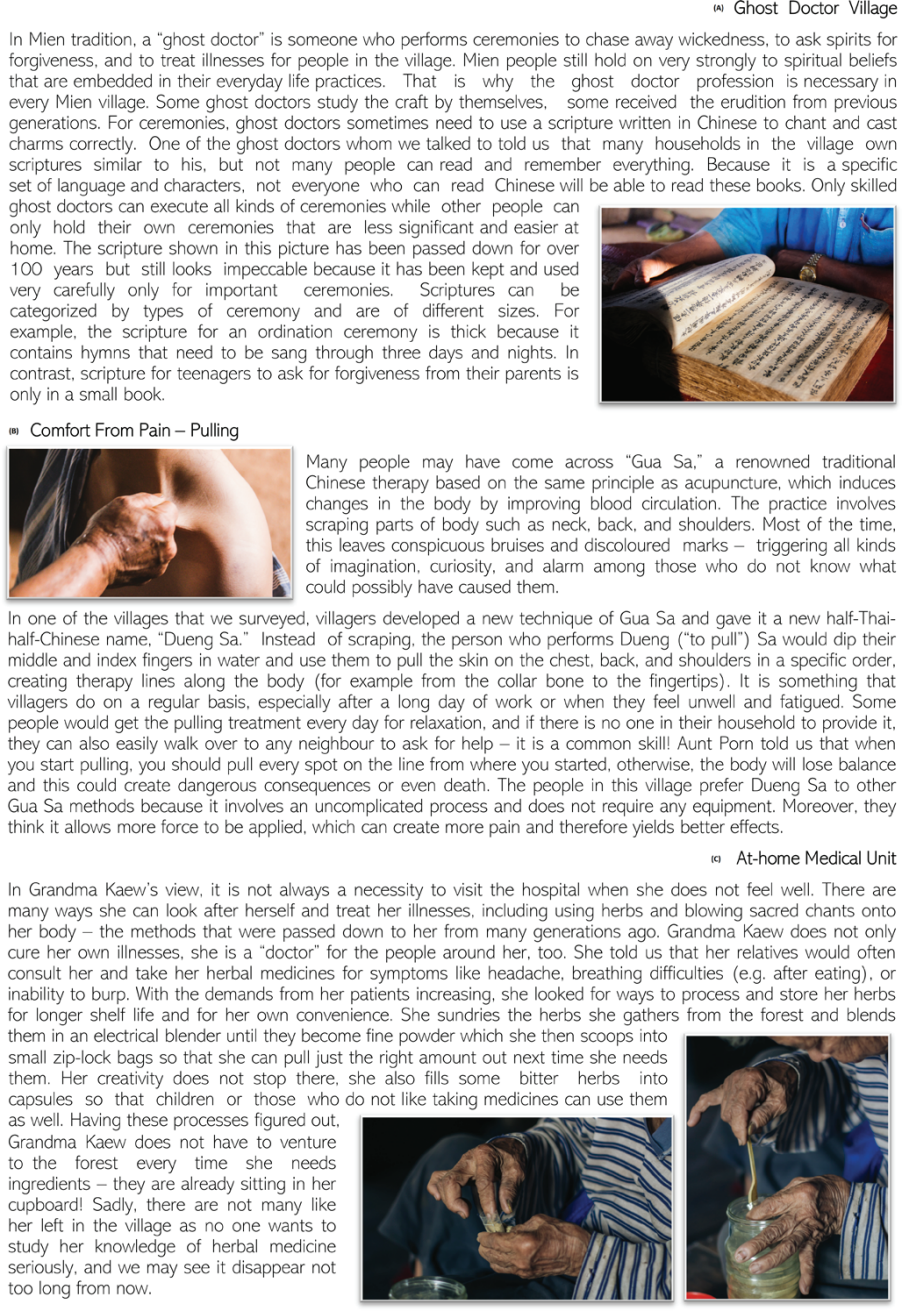
Tales of Treatment. Source: Tales of Treatment exhibition booklet.

Figure 3A presents such a narrative from a traditional ‘ghost doctor’ (a spiritual healer) in a Mien village. The tale told of sacred books of chants in traditional Chinese, which in their entirety were often only accessible to ghost doctors who learnt their craft over generations. However, minor chants and small ceremonies were not reserved exclusively to the ghost doctor—it was a common skill in the village, applied for instance when teenagers sought forgiveness from their parents. The reflections sparked by this tale among the research team were twofold: first, the boundaries of ‘treatment’ extended beyond our initial (biomedically shaped) conceptions of what the roles of a traditional healer and spiritual care might involve. How could practices like asking for forgiveness be incorporated into a standardised survey instrument on treatment seeking, and how might the omission of, for example, pastoral dimensions of care distort the representation of local realities? Second, the fluid interpretation of who was a ghost doctor in a village (ie, potentially everyone) undermined our initially binary distinction between the general population versus medical providers.

The second example from the Tales of Treatment exhibition involved a traditional treatment adapted from ‘*gua sa*’ (กัวซา), which was common in Thailand, China and Southeast Asia more broadly. Also known as *gua sha* in Chinese (刮痧), or ‘scraping’/‘coining’ in English,150 *gua sa* involved scraping the skin to stimulate blood circulation until bruises appear. The tale relayed in figure 3B told of local adaptations of this practice that involved pulling rather than scraping the skin—locally known as ‘*dueng sa*’ (ดึงซา). Like the spiritual chants in the previous narrative, *dueng sa* was a common skill among villagers, and its superior effectiveness over *gua sa* was explained by the pain it inflicted on the recipient. The insights generated by this tale did not only involve the local adaptation of international yet non-Western medical practices of *gua sha* and the (for us) unexpected interpretations of how people assessed the quality of *dueng sa*—very much unlike conventional Western interpretations of what ‘quality of care’ would entail. One of the main surprises from this story was also the idiosyncrasy of medical practice. Aunt Porn’s village performed a version of *gua sa* that was different from local medical practice in neighbouring villages. This begged the question, ‘What does “traditional healing” mean at all, and how can we usefully categorise it?’ (This is of course not a fundamentally new question as medical anthropology and medical humanities have been engaging with such topics for decades.)

The final narrative (figure 3C) related to the broader discourse of AMR as a global health issue. Grandma Kaew was among the last traditional healers in her village, applying knowledge passed down to her from generations ago. Fellow villagers received her herbal treatment for symptoms like headaches and indigestion, and steady demand had required her to process these herbs more efficiently. As she explored methods to store herbs for convenience and longer shelf life, she begun sun-drying them, blending them into fine powder and apportioning them into small zip-lock bags. She also filled bitter-tasting herbs like ‘*fah talai jone*’ (‘ฟ้าทะลายโจร’ or *andrographis paniculate*) into capsules so that children or patients who did not like taking medicines could use them as well.

The significance of Grandma Keaw’s story rested in the fact that modern Thai health policy had begun advocating, among others, the treatment of uncomplicated conditions like sore throats with traditional Thai herbal medicine. The purpose of this development had been to respond to healthcare providers ‘who feel pressured by patients’ expectations’ for antibiotics and therefore reduce the reliance on antibiotic treatment in human medicine.151 The tale of Grandma Keaw’s ‘at-home medical unit’ underlined the irony of this proposal: herbal and non-medicinal alternatives for antibiotic treatment had been practised for centuries, but were over the past decades crowded out increasingly by the modernisation of medicine.152, 153 On reflection, one could argue that modern medicine had sown the seeds of its own demise through the pharmaceuticalisation of care (ie, reducing the idea of healing to the transaction of capsules), and now depends for its survival on the traditional medicine that it had been displacing. At the same time, critical academic voices wonder whether the modern Thai health policy approach incorporating herbal medicine capsules does, yet again, reduce holistic traditional treatment to a transactional relationship. Although this might be a valid concern, Grandma Kaew herself had been producing and administering herbal medicines in capsules—for pragmatic reasons, and without obviously adhering to a biomedical agenda.

The practice of recording narratives alongside our survey therefore enabled our research team to perceive illness and treatment beyond the survey questionnaire. Examples of local medical practice challenged our conceptualisation of care in rural northern Thailand—for example, the dichotomy between population and healthcare providers, the spectrum of conditions that deserved a traditional healer’s attention and the fluidity of its performance—but it also added nuance to our understanding of modern health policy and its critiques in the context of AMR.

### Subcase 3 (dialogue): reactions and reflections from the photo exhibitions

‘Tales of Treatment’ was a mechanism to capture narratives from northern Thai villages, and to acquaint the urban public interested in photography, culture and alternative systems of medicine with this material. The overall >500 visitors across our four venues engaged enthusiastically with the exhibits, the stories and the exhibition hosts, typically spending 45–60 min at the exhibition. The dialogue between researchers and the public thereby enabled a further expansion of the understanding of the sociocultural context of AMR into areas and geographies that the research team had not initially considered.

Interactions between the research team and the exhibition attendees revealed how the exhibition stimulated reflection and recall of personal treatment histories. For example, some of our Thai audience, including those from northern Thailand, said that they had seen their parents or grandparents follow the practices shown in the photographic stories, but they had never experienced herbal or spiritual healing themselves. UK and US audience members related the content to the role of complementary medicine in their respective home countries, and drew parallels between Thailand and Western countries in terms of sensemaking about the body, illness and healing techniques.

Written feedback also suggested that the exhibition sparked reflection. Attendees related the content to their personal experiences growing up in families where modern medicine was unpopular (“*My dad never liked modern medicines so I’ve experienced* (traditional and alternative forms of healing) *a lot! Acupuncture, power therapy, psychotherapy,* […]”), or their encounters with traditional medicine in other Southeast Asian contexts (“[…] *In Vietnam, we have a practice called cao gió—very popular for ‘scratch*(ing) *out the wind’ from a cold/fever* […]”). Together with the attendees, we reflected yet further on cross-cultural comparisons of behaviour and possible research avenues about the co-evolution and global spread of drug resistance and local forms of healing.

During the latest iteration of the exhibition at Warwick Arts Centre, we collected more formal feedback in addition to verbal and guestbook testimonies. At a response rate of 32.9% (23 out of 70 visitors, all of whom were university students or staff), 95.7% agreed that they learnt ‘something new’ during the exhibition (100% of the responses agreed that the event was ‘worthwhile’). Among the explanations of what had been learnt, the attendees indicated, for example,

- ‘*“Alternative” treatments in other parts of the world*’;
- ‘*The popularity of using the supernatural*’;
- ‘*The interconnectedness of Thai, Chinese medicine*’;
- ‘*So much! In particular the pulling and pinching* (*gua sa*, *dueng sa*)’.

Yet, not everyone was equally impressed. A subset of the attendees in all exhibition sites also expressed doubts both about the content of the stories (eg, narratives about medicinal plants functioning as fever absorber) and the photographs themselves (eg, concerns about animal cruelty where ghost doctors used tiger claws during treatment). Specifically with regard to antibiotics and drug resistance, some attendees in Bangkok also enquired about the subject, behaving as if the team were medical specialists. Although such feedback and reflections only arose in conversation with the attendees rather than in writing, some also indicated that they had ‘*never realised how effective these treatments can be*’. The exhibition stated explicitly that its intention was not to advocate a particular treatment method nor to suggest the superiority of traditional healing—rather, to relay stories from the field. Nevertheless, we as hosts may have on occasion been misinterpreted as medical specialists, and some interpretations of the content may have potentially entailed unintended behavioural outcomes of the public engagement event.

## Discussion

### Summary

The case study exemplified that public engagement under the umbrella of the medical humanities—that is, not focused on instrumental awareness raising and community mobilisation—can complement and contribute to the understanding of local health practices and global health priorities. Our activities involved knowledge co-production, storytelling and dialogue between researchers and the public, and they emerged partly in response to the limitations of a health behaviour survey. Overall, the three subcases enabled:

- a better understanding of local conceptualisations of medicine (subcases 1, 2, 3);
- new insights into the social configuration of treatment seeking (subcases 1 and 2);
- for us otherwise invisible idiosyncrasies of traditional healing across villages in northern Thailand (subcase 2);
- new perspectives on the relationship between ‘the general population’ and ‘traditional healers’ (subcases 2 and 3);
- reflection on the relationship between modernity and tradition in AMR (subcase 2).

At the same time, not all points raised in the co-production workshops could be supported by our quantitative survey data, and participation in the exhibitions appeared to have created misleading impressions of our purpose and messages among a small group of attendees.154 Despite its seeming value for challenging thought and research in global health, we should therefore not underestimate the consequences of intervening in a social system through co-production and bi-directional communication—however well-meaning it might be.

Our case study contributes to the practice of public engagement in global health research as an important element of sustainable development and the empirical understanding of the sociocultural context of AMR as a global health priority. As opposed to mainstream community engagement activities in global health and AMR in particular,155 the case study suggested how medical humanities methods can help researchers to learn from their target populations instead of instrumentalising ‘engagement’ to change communities along biomedical ideals. The importance of bi-directional communication highlighted in our work indicated that global health researchers indeed *require* local inputs to formulate hypotheses and ground analytical categories, and also to define the research problem itself—similar to arguments surrounding the practice of patient and public involvement in Western medical research.156, 157 By depicting new and for biomedical researchers often invisible subjective truths, our case also supports positions in the medical humanities that knowledge co-production, stories and dialogue based on artistic production can yield new and practically important perspectives on complex problems158, 159—for example, healing and the nature of health systems—that have bearing on AMR. One often neglected aspect of healing is for instance the role of spirituality,160 as the tales of treatment (subcase 2) have powerfully brought to the fore (spirituality may play a role in considering non-pharmaceutical solutions for population health and well-being, eg, in the form of pastoral support and meaning-making). At the same time, the documented risks of the unintended consequences of engagement also expand the recent argument by Abimbola161 (in the context of community health committees) to steer global health researchers and practitioners away from an unrealistically optimistic ‘a priori bias’ in community engagement.

The insights provided by our workshop participants (subcase 1) and exhibition attendees (subcase 3) further added to debates and empirical knowledge in the field of AMR. For example, knowledge co-production in the workshops highlighted the varied relationship between antibiotic conceptions and attitudes towards over-the-counter purchases and related to the literature on language and local conceptions of antimicrobials.162, 163 Other locally grounded research hypotheses demonstrated how antibiotic usage differed across generations, which contributed to understanding the determinants of antibiotic use and the values that underlie antibiotic choices in Thailand and other LMICs.164–166 In addition, biomedical writing often portrays traditional healers as an unqualified source of antimicrobials or as a healthcare solution that could delay access to biomedically trained healthcare providers.167, 168 Rather than pitching traditional against formal healthcare, stories of healing and treatment (subcase 2) demonstrated the fluidity of traditional healing in Chiang Rai and, together with the dialogue with urban publics, enabled reflections on its relationship to AMR. Considering that ‘AMR is understood as a threat to health, to economies, to security and to modernity itself’,169 traditional healing may ironically play a role in ‘saving’ modern medicine by limiting dependence on pharmaceutical treatment in the case of uncomplicated minor ailments such as muscle pains or sore throats (as the tale of Grandma Kaew in subcase 2 illustrated). Overall, the inputs from workshop participants, traditional healers and exhibition attendees in our project challenged assumptions and expectations among the international research team, helping to expand understanding incrementally and to challenge geographically and disciplinarily defined hierarchies of knowledge in global health research.

### Costs and risks of knowledge co-production

Overall, our analysis suggested that there were complementarities between the co-production of knowledge on the one hand, and the data collection methods and the interpretation of health behaviour research on the other hand. However, these activities also produced costs and risky outcomes that we discuss briefly in this section.

Short of immersive ethnographic research, cross-sectional qualitative research could have similarly helped to generate knowledge about local behaviours and medicine use, and to inform the development of a structured questionnaire. Qualitative pretesting of the survey instruments—for instance, through cognitive interviewing170—can help uncover unforeseen categories and refine quantitative data collection as well, although this often happens at a stage when research design and hypotheses are already relatively fixed. We applied both these techniques in this study, but the workshop setup helped to complement these qualitative approaches. Although activities like medicine pile sorting are not specific to a workshop setting and could in principle be also incorporated into semi-structured interviews and focus group discussions (‘participatory’ methods like pile sorting exercises have long been incorporated in development survey research171), the wide range of media and activities employed during the workshops helped generate a more open and engaging atmosphere and a greater degree of bi-directional knowledge exchange than could be achieved in the more structured data collection settings of face-to-face interviews or focus group discussions. The monetary costs of the workshops themselves amounted to £450 per workshop for consumables and eight facilitating staff plus approximately £3000 for consumables and staff costs for the development and trialling of the workshop format.

Similarly, gathering and exhibiting photographic narratives from our field sites was an opportunity for the project to cultivate and benefit from the talent of the Thai research team, and to learn about healing and treatment from the perspective of local residents. The narratives enabled us to explore perspectives that especially the non-Thai project collaborators would not have considered otherwise. The visual component of the narratives thereby offered additional space for reflection compared with, for instance, a solely text-based semi-structured interview, and it opened a pathway to engaging the broader public interested in photography, culture and traditional healing in our project. The latest exhibition at Warwick Arts Centre also paved the way for closer collaboration between the research team and the creative industry.

However, knowledge co-production through visual methods and storytelling served primarily a supplementary purpose in our project—for our research objectives, it would have not have sufficed as an alone-standing research and knowledge production method (which comes with its own methodological and ethical challenges, compounded by interdisciplinary frictions in ethical review committees172–176). As the feedback from the photo exhibitions showed, presenting health-related practices could also potentially influence people’s health behaviour even if the research team explicitly distanced themselves from advocating any particular practice. The collection and preparation of the material and hosting the four photo exhibitions also required a budget of approximately £8000.

These costs and risks mean that knowledge co-production has to be weighed against alternative qualitative and quantitative modes of generating global health knowledge. As a complement to conventional research methods, however, they can usefully inform a project during its design phase, aid the interpretation of its results, and make the dissemination of its findings more effective. The costs and risks of these methods should therefore be assessed on the basis of complementing conventional global health research.

### Limitations

The primary limitation of this research was that the co-production activities did not involve an independent evaluation. Being embedded in the research and positioning ourselves as ‘learners’ vis-à-vis villagers and the public prevented the research team from carrying out a formal independent assessment of the consequences of the activities. A parallel research team not involved in the project or its design would have ideally worked alongside our group to add additional depth on the unintended consequences and potential (and actual) benefits and harms arising from the co-production activities. Despite our best attempts to be mindful of alternative interpretations of our work and the negative outcomes of the activities, there remained thus a residual risk that our position as social researchers invested in this project unconsciously biased us towards a particular interpretation of the data and participants’ responses.

The mixed insights from the quantitative analysis further indicated the shortcomings of using a prespecified survey instrument to assess locally emerging research hypotheses. Especially where the quantitative findings did not support the hypotheses, the question remained whether this was because the hypothesis could not be supported, or because the questionnaire and research design were not suitable to investigate the respective point. Although the questionnaire was developed with the help of prior qualitative research, field pilots and cognitive interviewing, the new insights provided by the participatory workshops would have required further iterations to accommodate workshop participants’ inputs.

An additional limitation of the scope of this case study is that the public engagement team comprised social scientists and no medical specialists. As a result, our focus is confined to sociocultural aspects of AMR, in particular local knowledge and intersubjective truths on health systems and healing. These aspects are important to understand the phenomenon of (and responses to) drug resistance more broadly, but we cannot make claims about biological mechanisms, clinical dimensions or the direct contribution of people’s behaviours to the development of AMR.

## Conclusion

Against the backdrop of growing critiques of neocolonial practices and hierarchical relationships in global health research—resembling the modernisation paradigm in international development—we asked, ‘*Can medical humanities approaches challenge hierarchies and promote engagement in global health research on antimicrobial resistance?*’ Our underlying objective was to demonstrate how the inclusion of medical humanities methods in public engagement activities can open up new and locally grounded perspectives for thinking about the sociocultural context of AMR and its related topics of medicine use and health systems. To this end, we studied the case of public engagement with research in Chiang Rai province, northern Thailand, that co-produced knowledge through participatory workshops, collected photographic and oral narratives of healing and treatment and engaged in public dialogue through the display of these ‘Tales of Treatment’. The case study illustrated that locally grounded hypotheses and the production, reflection and display of photographic narratives could challenge our own assumptions as health behaviour researchers, and offered new perspectives on global health debates in AMR. The short and perhaps unsurprising answer to the research question therefore is ‘yes,’ medical humanities methods can make public engagement a vehicle to challenge external assumptions of illness and treatment and potentially undermine entrenched hierarchies of knowledge. Creative forms of expression and participatory means can facilitate this co-production and mutual exchange between local populations and researchers. While the tension between local and global knowledge (and between ‘the traditional’ and ‘the modern’) remains a challenge in global health research and practice, it also provides space in which creative methods can flourish.177

However, a tension also remains between the benefits of co-producing knowledge and the risk of unintended consequences from public engagement and the presence of external research teams. Ours is not the first study to problematise hazards of potentially detrimental interpretations of narratives and arts-based engagement with the public,178, 179 misinterpretation of roles and competences of researchers180 or issues of preproducing hierarchical relationships to the point of oppressing local communities.181 The knowledge to evaluate such mixed consequences of public engagement and participatory research is yet limited and requires further methodological research.182–185 Once evaluation frameworks and guidelines have been established, varied applications of process, ex post and impact evaluation (both qualitative and quantitative) would enable us to map the consequences of knowledge co-production and to assess their costs and benefits more comprehensively and pragmatically—even if the costs of an evaluation itself mean that such assessments can only be conducted on a sample of research projects.

Overall, our analysis leads us to conclude that knowledge co-production and medical-humanities-informed forms of public engagement should become standard secondary objectives of global health research to prevent misrepresentation of local realities and to more effectively ground the interpretations of its findings in the local context. One precondition of this strategy to succeed is to frame global health research more actively as a learning exercise and embed the agenda to ‘decolonise’ global health more firmly in research education and international health policy circles. An international commission—led by interdisciplinary researchers from LMICs—could further legitimise this practice by establishing formal ethical guidelines for global health research to be more receptive to local voices, rather than merely instrumentalising the rhetoric of public engagement for public health interventions.

## Data Availability

Data are available in a public repository via safeguarded access on the UK Data Service via the following reference: Haenssgen, M. J., P. Ariana, H. F. L. Wertheim, R. C. Greer, C. Jones, Y. Lubell, *et al*. 2019. Antibiotics and activity spaces: rural health behaviour survey in Northern Thailand and Southern Laos 2017-2018 (data set). Colchester: UK Data Service. doi: 10.5255/UKDA-SN-853658.

## Notes

1. R. C Keller (2006). "Geographies of power, legacies of mistrust: colonial medicine in the global present." *Historical Geography* no. 34:26-48.

2. Bridget Pratt et al. (2018). "Exploring the ethics of global health research priority-setting." *BMC Medical Ethics* no. 19 (94). doi: 10.1186/s12910-018-0333-y

3. Richard Horton (2013). "Offline: is global health neocolonialist?" *The Lancet* no. 382 (9906):1690. doi: 10.1016/S0140-6736(13)62379-X

4. Anonymous (2019). "Editorial: break with tradition: the World Health Organization’s decision about traditional Chinese medicine could backfire." *Nature* no. 570:5.

5. S. S Amrith (2006). *Decolonizing international health: India and Southeast Asia, 1930–65*. London: Palgrave Macmillan.

6. Arturo Escobar and A Escobar (1984). "Discourse and power in development: Michel Foucault and the relevance of his work to the third world." *Alternatives* no. 10 (3):377-400. doi: 10.1177/030437548401000304

7. UNDG (2013). *A million voices: the world we want: a sustainable future with dignity for all*. New York, NY: United Nations Development Group.

8. WHO (2019). *Speech by the Director-General: transforming for impact* 2019 (cited 10 March 2019). Available from: https://www.who.int/dg/speeches/detail/transforming-for-impact.

9. R. C Keller (2006). Geographies of power, legacies of mistrust: colonial medicine in the global present.

10. Mishal S Khan et al. (2019). Durrance-Bagale, H. Legido-Quigley "‘LMICs as reservoirs of AMR’: a comparative analysis of policy discourse on antimicrobial resistance with reference to Pakistan." *Health Policy and Planning* no. 34 (3):178–187. doi: 10.1093/heapol/czz022

11. Clare I R Chandler (2019). "Current accounts of antimicrobial resistance: stabilisation, individualisation and antibiotics as infrastructure." *Palgrave Communications* no. 5 (1):53. doi: 10.1057/s41599-019-0263-4

12. In the area of antimicrobial use for human health, other problem areas include, for example, public hygiene and disease prevention, regulated access to medicines, disease diagnosis, or market conditions for the development of new antimicrobials. The Review on Antimicrobial Resistance (2016). *Tackling drug-resistant infections globally: final report and recommendations*. London: The UK Prime Minister, WHO (2015b). *Global action plan on antimicrobial resistance*. Geneva: World Health Organization, Conan MacDougall and Ron E Polk (2005). "Antimicrobial stewardship programs in health care systems." *Clinical Microbiology Reviews* no. 18 (4):638-656. doi: 10.1128/CMR.18.4.638-656.2005.

13. The Review on Antimicrobial Resistance. *Tackling drug-resistant infections globally: final report and recommendations*.

14. WHO, *Global action plan on antimicrobial resistance*.

15. Maria R Gualano et al. (2015). "General population's knowledge and attitudes about antibiotics: a systematic review and meta-analysis." *Pharmacoepidemiology and Drug Safety* no. 24 (1):2-10. doi: 10.1002/pds.3716

16. H Haak and A. Radyowijati (2010). "Determinants of antimicrobial use: poorly understood, poorly researched." In *Antimicrobial resistance in developing countries*, edited by Sosa, Byarugaba, Amábile-Cuevas, Hsueh, Kariuki and Okeke, 283-300. New York, NY: Springer.

17. These problems persist despite encouraging trends. For example, the field is becoming increasingly multidisciplinary through the involvement of several United Nations agencies alongside WHO in governing AMR, and AMR policy narratives are slowly broadening the hitherto hyper-individualised and behaviour change focus of global action plans. Connor Rochford et al. (2018). "Global governance of antimicrobial resistance." *The Lancet* no. 391 (10134):1976-1978. doi: 10.1016/S0140-6736(18)31117-6, WHO, FAO, and OIE (2018). *Monitoring global progress on addressing antimicrobial resistance: analysis report of the second round of results of AMR country self-assessment survey 2018*. Geneva: World Health Organization, Food and Agriculture Organization of the United Nations and World Organisation for Animal Health (OIE), WHO (2017). *Antimicrobial Resistance Behaviour Change first informal technical consultation, 6-7 November, 2017 Château de Penthes, Geneva: Meeting Report*. Geneva: World Health Organization, Elise Klein and China Mills (2017). "Psy-expertise, therapeutic culture and the politics of the personal in development." *Third World Quarterly* no. 38 (9):1990-2008. doi: 10.1080/01436597.2017.1319277

18. Emma R M Cohen et al. (2008). "Public engagement on global health challenges." *BMC Public Health* no. 8 (168). doi: 10.1186/1471-2458-8-168

19. B Hamlyn et al. (2015).*Factors affecting public engagement by researchers: a study on behalf of a consortium of UK public research funders*. London: TNS

20. Research Councils UK (2011) *Concordat for engaging the public with research*. Research Councils UK: Swindon.

21. C Wilson, P. Manners, and S. Duncan (2014). *Building an engaged future for UK higher education: full report from the Engaged Futures consultation*. Bristol: National Co-ordinating Centre for Public Engagement.

22. Also referred to as ‘community engagement’, ‘patient and public involvement’ (PPI) in research, or in some instances also as participatory research. S. Staniszewska et al. (2017). "GRIPP2 reporting checklists: tools to improve reporting of patient and public involvement in research." *Research Involvement and Engagement* no. 3 (13). doi: 10.1186/s40900-017-0062-2, Jo Brett et al. (2014). "Mapping the impact of patient and public involvement on health and social care research: a systematic review." *Health Expectations* no. 17 (5):637-650. doi: 10.1111/j.1369-7625.2012.00795.x, Paulina O Tindana et al. (2007). "Grand challenges in global health: community engagement in research in developing countries." *PLOS Medicine* no. 4 (e273). doi: 10.1371/journal.pmed.0040273, F Darroch and A. Giles (2014). "Decolonizing health research: community-based participatory research and postcolonial feminist theory." *Canadian Journal of Action Research* no. 15 (3):22-36.

23. J Redfern et al. (2018). "Spreading the message of antimicrobial resistance: a detailed account of a successful public engagement event." *FEMS Microbiology Letters* no. 365 (16). doi: 10.1093/femsle/fny175

24. Victoria Jane Hume et al. (2018). "Biomedicine and the humanities: growing pains." *Medical Humanities* no. 44 (4):230-238. doi: 10.1136/medhum-2018-011481

25. Astrid Treffry-Goatley et al. (2018). Ibid. "Community engagement with HIV drug adherence in rural South Africa: a transdisciplinary approach." 239-246. doi: 10.1136/medhum-2018-011474

26. L Jordanova (2014). "Medicine and the visual arts." In *Medicine, health and the arts: approaches to medical humanities*, edited by Bates, Bleakley and Goodman, 41-63. Abingdon: Routledge.

27. Angela Ross Perfetti (2018). "Fate and the clinic: a multidisciplinary consideration of fatalism in health behaviour." *Medical Humanities* no. 44 (1):59-62. doi: 10.1136/medhum-2017-011319

28. Devan Stahl et al. (2016). "Seeing illness in art and medicine: a patient and printmaker collaboration." Ibid. no. 42 (3):155-159. doi: 10.1136/medhum-2015-010838

29. Jonatan Wistrand and J Wistrand (2017). "When doctors are patients: a narrative study of help-seeking behaviour among addicted physicians." Ibid. no. 43 (1):19-23. doi: 10.1136/medhum-2016-011002

30. T. R Cole, N. S. Carlin, and R. A. Carson (2015). *Medical humanities: an introduction*. New York, NY: Cambridge University Press.

31. Daniel Holman and Erica Borgstrom (2016). "Applying social theory to understand health-related behaviours." *Medical Humanities* no. 42 (2):143-145. doi: 10.1136/medhum-2015-010688

32. Hume, *et al.*, Biomedicine and the humanities: growing pains.

33. A Carusi (2016). "Modelling systems biomedicine: intertwinement and the 'real'." In *The Edinburgh companion to the critical medical humanities*, edited by Whitehead, Woods, Atkinson, Macnaughton and Richards, 50-65. Edinburgh: Edinburgh University Press.

34. Jordanova, Medicine and the visual arts.

35. Stahl and Stahl, Seeing illness in art and medicine: a patient and printmaker collaboration.

36. William Viney et al. (2015). "Critical medical humanities: embracing entanglement, taking risks." Ibid. no. 41 (1):2-7. doi: 10.1136/medhum-2015-010692

37. J Cole and S. Gallagher (2016). "Narrative and clinical neuroscience: can phenomenologically informed approaches and empirical work cross-fertilise?" In *The Edinburgh companion to the critical medical humanities*, edited by Whitehead, Woods, Atkinson, Macnaughton and Richards, 377-394. Edinburgh: Edinburgh University Press.

38. J Macnaughton and H. Carel (2016). Ibid."Breathing and breathlessness in clinic and culture: using critical medical humanities to bridge an epistemic gap." In, 294-309.

39. P J Pelto and G H Pelto (1997). 1997. "Studying knowledge, culture, and behavior in applied medical anthropology." *Medical Anthropology Quarterly* no. 11 (2):147-163.

40. Lindsay Prior (2003) "Belief, knowledge and expertise: the emergence of the lay expert in medical sociology." *Sociology of Health & Illness* no. 25 (3):41-57. doi: 10.1111/1467-9566.00339

41. E Oliveira and J. Vearey (2018). "Making research and building knowledge with communities: examining three participatory visual and narrative projects with migrants who sell sex in South Africa." In *Creating social change through creativity: anti-oppressive arts-based research methodologies*, edited by Capous-Desyllas and Morgaine, 265-287. Cham: Springer.

42. Komatra Chuengsatiansup and Wirun Limsawart (2019). "Tuberculosis in the borderlands: migrants, microbes and more-than-human borders." *Palgrave Communications* no. 5 (1):31. doi: 10.1057/s41599-019-0239-4

43. R Garden (2014). "Social studies: the humanities, narrative, and the social context of the patient-professional relationship." In *Health humanities reader*, edited by Jones, Wear, Friedman and Pachucki, 127-137. New Brunswick, NJ: Rutgers University Press.

44. Holman and Borgstrom, Applying social theory to understand health-related behaviours.

45. Claas Kirchhelle (2018). "Pharming animals: a global history of antibiotics in food production (1935–2017)." *Palgrave Communications* no. 4 (96). doi: 10.1057/s41599-018-0152-2

46. Hannah Landecker (2019). "Antimicrobials before antibiotics: war, peace, and disinfectants." Ibid. no. 5 (1):45. doi: 10.1057/s41599-019-0251-8

47. Sue Walker (2019). Ibid."Effective antimicrobial resistance communication: the role of information design." 24. doi: 10.1057/s41599-019-0231-z

48. Pelto and Pelto, Studying knowledge, culture, and behavior in applied medical anthropology.

49. May Sudhinaraset et al. (2013). "What is the role of informal healthcare providers in developing countries? A systematic review." *PLoS ONE* no. 8 (2):e54978. doi: 10.1371/journal.pone.0054978

50. Viroj Tangcharoensathien, Sunicha Chanvatik, and Angkana Sommanustweechai (2018). "Complex determinants of inappropriate use of antibiotics." *Bulletin of the World Health Organization* no. 96 (2):141-144. doi: 10.2471/BLT.17.199687

51. WHO (2015a). *Antibiotic resistance: multi-country public awareness survey*. Geneva: World Health Organization.

52. WHO, *Antibiotic resistance: multi-country public awareness survey*, 42.

53. Gualano, *et al.* General population's knowledge and attitudes about antibiotics: a systematic review and meta-analysis.

54. Edward A Belongia et al. (2002). "Antibiotic use and upper respiratory infections: a survey of knowledge, attitudes, and experience in Wisconsin and Minnesota." *Preventive Medicine* no. 34 (3):346-352. doi: 10.1006/pmed.2001.0992

55. Miao Yu et al. (2014). "Knowledge, attitudes, and practices of parents in rural China on the use of antibiotics in children: a cross-sectional study." *BMC Infectious Diseases* no. 14 (112). doi: 10.1186/1471-2334-14-112

56. Abdelmoneim Ismail Awad and Esraa Abdulwahid Aboud (2015). "Knowledge, attitude and practice towards antibiotic use among the public in Kuwait." *PLoS ONE* no. 10 (2):e0117910. doi: 10.1371/journal.pone.0117910

57. Chandler, Current accounts of antimicrobial resistance: stabilisation, individualisation and antibiotics as infrastructure.

58. Jie Chang et al. (2018). "Non-prescription use of antibiotics among children in urban China: a cross-sectional survey of knowledge, attitudes, and practices." *Expert Review of Anti-infective Therapy* no. 16 (2):163-172. doi: 10.1080/14787210.2018.1425616

59. Gualano, *et al.* General population's knowledge and attitudes about antibiotics: a systematic review and meta-analysis.

60. A R McCullough et al. (2016). "A systematic review of the public's knowledge and beliefs about antibiotic resistance." *Journal of Antimicrobial Chemotherapy* no. 71 (1):27-33. doi: 10.1093/jac/dkv310

61. Abel Santiago Muri-Gama, Albert Figueras, and Silvia Regina Secoli (2018). "Inappropriately prescribed and over-the-counter antimicrobials in the Brazilian Amazon Basin: we need to promote more rational use even in remote places." *PLoS ONE* no. 13 (e0201579). doi: 10.1371/journal.pone.0201579

62. A Launiala (2009). "How much can a KAP survey tell us about people's knowledge, attitudes and practices? Some observations from medical anthropology research on malaria in pregnancy in Malawi." *Anthropology Matters* no. 11 (1).

63. Pamela Das et al. (2016). "Antibiotics: achieving the balance between access and excess." *The Lancet* no. 387 (10014):102-104. doi: 10.1016/S0140-6736(15)00729-1

64. C Olivier et al. (2010). "Containing global antibiotic resistance: ethical drug promotion in the developing world." In *Antimicrobial resistance in developing countries*, edited by Sosa, Byarugaba, Amábile-Cuevas, Hsueh, Kariuki and Okeke, 505-524. New York, NY: Springer.

65. Chandler, Current accounts of antimicrobial resistance: stabilisation, individualisation and antibiotics as infrastructure.

66. Chandler, Current accounts of antimicrobial resistance: stabilisation, individualisation and antibiotics as infrastructure.

67. Steve Hinchliffe, Andrea Butcher, and Muhammad Meezanur Rahman (2018). "The AMR problem: demanding economies, biological margins, and co-producing alternative strategies." Ibid. no. 4 (142). doi: 10.1057/s41599-018-0195-4

68. Chuengsatiansup and Limsawart, Tuberculosis in the borderlands: migrants, microbes and more-than-human borders.

69. Khan, *et al*, ‘LMICs as reservoirs of AMR’: a comparative analysis of policy discourse on antimicrobial resistance with reference to Pakistan.

70. Didier Wernli et al. (2017). "Mapping global policy discourse on antimicrobial resistance." *BMJ Global Health* no. 2 (e000378). doi: 10.1136/bmjgh-2017-000378

71. Nancy J Hawkings, Fiona Wood, and Christopher C Butler (2007). "Public attitudes towards bacterial resistance: a qualitative study." *Journal of Antimicrobial Chemotherapy* no. 59 (6):1155-1160. doi: 10.1093/jac/dkm103

72. McCullough, *et al.* A systematic review of the public's knowledge and beliefs about antibiotic resistance.

73. Muri-Gama, *et al.* Inappropriately prescribed and over-the-counter antimicrobials in the Brazilian Amazon Basin: we need to promote more rational use even in remote places.

74. David G Allison et al. (2017). "Antibiotic resistance awareness: a public engagement approach for all pharmacists." *International Journal of Pharmacy Practice* no. 25 (1):93-96. doi: 10.1111/ijpp.12287

75. Mark Davis et al. (2018). "Understanding media publics and the antimicrobial resistance crisis." *Global Public Health* no. 13 (9):1158-1168. doi: 10.1080/17441692.2017.1336248

76. Simon J Howard et al. (2013). "Antibiotic resistance: global response needed." *The Lancet Infectious Diseases* no. 13 (12):1001-1003. doi: 10.1016/S1473-3099(13)70195-6

77. Renly Lim et al. (2016). "Village drama against malaria." *The Lancet* no. 388 (10063):2990. doi: 10.1016/S0140-6736(16)32519-3

78. Deborah Nyirenda et al. (2018). "Public engagement in Malawi through a health-talk radio programme ‘Umoyo nkukambirana’: a mixed-methods evaluation." *Public Understanding of Science* no. 27 (2):229-242. doi: 10.1177/0963662516656110

79. Redfern, *et al.* Spreading the message of antimicrobial resistance: a detailed account of a successful public engagement event.

80. Sungjong Roh et al. (2018). "Public understanding of One Health messages: the role of temporal framing." *Public Understanding of Science* no. 27 (2):185-196. doi: 10.1177/0963662516670805

81. Tindana, *et al.*, Grand challenges in global health: community engagement in research in developing countries.

82. Mpoe Johannah Keikelame and Leslie Swartz (2019). "Decolonising research methodologies: lessons from a qualitative research project, Cape Town, South Africa." *Global Health Action* no. 12 (1):1561175. doi: 10.1080/16549716.2018.1561175

83. Keymanthri Moodley and Shenuka Singh (2016). "“It’s all about trust”: reflections of researchers on the complexity and controversy surrounding biobanking in South Africa." *BMC Medical Ethics* no. 17 (57). doi: 10.1186/s12910-016-0140-2

84. Seye Abimbola (2020). "Beyond positive a priori bias: reframing community engagement in LMICs (epub ahead of print)." *Health Promotion International*. doi: 10.1093/heapro/daz023

85. Keikelame and Swartz, Decolonising research methodologies: lessons from a qualitative research project, Cape Town, South Africa.

86. Kenneth M Boyd (2000). "Disease, illness, sickness, health, healing and wholeness: exploring some elusive concepts." *Medical Humanities* no. 26 (1):9-17. doi: 10.1136/mh.26.1.9

87. Hume, *et al.*, Biomedicine and the humanities: growing pains.

88. I Bamforth (2000). "Kafka's uncle: scenes from a world of trust infected by suspicion." Ibid. no. 26 (2):85-91. doi: 10.1136/mh.26.2.85

89. Wistrand, When doctors are patients: a narrative study of help-seeking behaviour among addicted physicians.

90. Garden, Social studies: the humanities, narrative, and the social context of the patient-professional relationship.

91. A Harpin (2016). "Broadmoor performed: a theatrical hospital." In *The Edinburgh companion to the critical medical humanities*, edited by Whitehead, Woods, Atkinson, Macnaughton and Richards, 577-595. Edinburgh: Edinburgh University Press.

92. Jordanova, Medicine and the visual arts.

93. Stahl and Stahl, Seeing illness in art and medicine: a patient and printmaker collaboration.

94. K G Sweeney et al. (2001). "A comparison of professionals' and patients' understanding of asthma: evidence of emerging dualities?" Ibid. no. 27 (1):20-25. doi: 10.1136/mh.27.1.20

95. Treffry-Goatley, *et al.*, Community engagement with HIV drug adherence in rural South Africa: a transdisciplinary approach.

96. R. J Hester (2016). "Culture in medicine: an argument against competence." In *The Edinburgh companion to the critical medical humanities*, edited by Whitehead, Woods, Atkinson, Macnaughton and Richards, 541-558. Edinburgh: Edinburgh University Press.

97. L Jerke, M. Prendergast, and W. Dobson (2018). "Smoking cessation in mental health communities: a living newspaper applied theatre project." In *Creating social change through creativity: anti-oppressive arts-based research methodologies*, edited by Capous-Desyllas and Morgaine, 171-186. Cham: Springer.

98. Sweeney, *et al.* A comparison of professionals' and patients' understanding of asthma: evidence of emerging dualities?

99. S Switzer (2018). "What’s in an image? Towards a critical and interdisciplinary reading of participatory visual methods." In *Creating social change through creativity: anti-oppressive arts-based research methodologies*, edited by Capous-Desyllas and Morgaine, 189-207. Cham: Springer.

100. Cole and Gallagher, Narrative and clinical neuroscience: can phenomenologically informed approaches and empirical work cross-fertilise?, 378.

101. Cole, *et al*. *Medical humanities: an introduction*.

102. J Herman (2001). "Medicine: the science and the art." *Medical Humanities* no. 27 (1):42-46. doi: 10.1136/mh.27.1.42

103. ^[^Viney, *et al.* Critical medical humanities: embracing entanglement, taking risks.

104. R. K Yin (2003). *Case study research: design and methods*. Thousand Oaks, CA: Sage.

105. Marco J Haenssgen et al. (2018)

106. S. L Gilman (2015). *Illness and image: case studies in the medical humanities*. New York, NY: Taylor & Francis.

107. HarbarthM Haughton (2018) . *Staging trauma: bodies in shadow*. London: Palgrave Macmillan.

108. S Hodge, J Robinson, and P Davis (2007). "Reading between the lines: the experiences of taking part in a community reading project." *Medical Humanities* no. 33 (2):100-104. doi: 10.1136/jmh.2006.000256

109. Hume, *et al.* Biomedicine and the humanities: growing pains.

110. Saam Idelji-Tehrani and Muna Al-Jawad (2019). "Exploring gendered leadership stereotypes in a shared leadership model in healthcare: a case study." Ibid. no. 45:388-398. doi: 10.1136/medhum-2018-011517

111. Suze M P J Jans et al. (2012). "A case study of haemoglobinopathy screening in the Netherlands: witnessing the past, lessons for the future." *Ethnicity & Health* no. 17 (3):217-239. doi: 10.1080/13557858.2011.604126

112. Hume, *et al.*, Biomedicine and the humanities: growing pains.

113. Cole and Gallagher, Narrative and clinical neuroscience: can phenomenologically informed approaches and empirical work cross-fertilise?

114. Macnaughton and Carel, Breathing and breathlessness in clinic and culture: using critical medical humanities to bridge an epistemic gap.

115. Pelto and Pelto, Studying knowledge, culture, and behavior in applied medical anthropology.

116. Prior, Belief, knowledge and expertise: the emergence of the lay expert in medical sociology.

117. Gilman, *Illness and image: case studies in the medical humanities*.

118. Cole and Gallagher, Narrative and clinical neuroscience: can phenomenologically informed approaches and empirical work cross-fertilise?

119. Macnaughton and Carel, Breathing and breathlessness in clinic and culture: using critical medical humanities to bridge an epistemic gap.

120. C Teddlie and A. Tashakkori (2009). *Foundations of mixed methods research: integrating quantitative and qualitative approaches in the social and behavioral sciences*. Thousand Oaks, CA: Sage.

121. Macnaughton and Carel, Breathing and breathlessness in clinic and culture: using critical medical humanities to bridge an epistemic gap.

122. Gian Luca Barbieri et al. (2016). "Imagination in narrative medicine." *Journal of Child Health Care* no. 20 (4):419-427. doi: 10.1177/1367493515625134

123. Treffry-Goatley, *et al.* Community engagement with HIV drug adherence in rural South Africa: a transdisciplinary approach.

124. WHO (2016). *World Antibiotic Awareness Week: 2016 campaign toolkit*. Geneva: World Health Organization.

125. Across the three villages, 67% of the workshop attendees were female and the average age of the attendees was 44 years (range: 18 to 81 years; based on subsequently collected survey data).

126. Nutcha Charoenboon et al. (2019)

127. We thank an anonymous reviewer for highlighting the potential hazards of reproducing hierarchies through methods intended to challenge them in the first place.

128. The research was reviewed and approved by the University of Oxford Tropical Research Ethics Committee (Ref. OxTREC 528-17), and it received local ethical approval in Thailand from the Mae Fah Luang University Research Ethics Committee on Human Research (Ref. REH 60099). The service evaluation of the photo exhibition involved anonymised data collection and received a waiver for ethical approval from the University of Warwick Humanities & Social Sciences Research Ethics Committee (HSSREC). However, all evaluation form respondents explicitly consented to the data being reported in research publications.

129. Marco J Haenssgen et al. (2018)

130. National Statistical Office (2012). *The 2010 population and housing census: Changwat Chiang Rai*. Bangkok: National Statistical Office.

131. Data on the individual level would entail duplication of observations should both census survey rounds be included. Step-level data were aggregated on the illness level for analysis.

132. Claire Charlotte McKechnie (2014). "Anxieties of communication: the limits of narrative in the medical humanities." *Medical Humanities* no. 40 (2):119-124. doi: 10.1136/medhum-2013-010466

133. Carusi, Modelling systems biomedicine: intertwinement and the 'real'.

134. Garden, Social studies: the humanities, narrative, and the social context of the patient-professional relationship.

135. Emma Sacks et al. (2018). "Beyond the building blocks: integrating community roles into health systems frameworks to achieve health for all." *BMJ Global Health* no. 3 (Suppl. 3):e001384. doi: 10.1136/bmjgh-2018-001384

136. Sudhinaraset, *et al.* What is the role of informal healthcare providers in developing countries? A systematic review.

137. G Bloom et al. (2015). *Addressing resistance to antibiotics in pluralistic health systems*. Brighton: University of Sussex

138. WHO (2007). *Strengthening health systems to improve health outcomes: WHO’s framework for action*. Geneva: World Health Organization.

139. Jordanova, Medicine and the visual arts.

140. Macnaughton and Carel, Breathing and breathlessness in clinic and culture: using critical medical humanities to bridge an epistemic gap.

141. A Bleakley (2014). Ibid. "Towards a 'critical medical humanities'." In, 17-26.

142. Hume, *et al.*, Biomedicine and the humanities: growing pains.

143. Nutcha Charoenboon et al. (2019)

144. Marco Haenssgen et al. (2018)

145. WHO, *World Antibiotic Awareness Week: 2016 campaign toolkit*.

146. The questionnaire did so by showing all survey respondents three images of common antibiotic capsules being used in Chiang Rai (green-blue: amoxicillin; red-black: cloxacillin; white-blue: azithromycin—see questionnaire page 10 in the online supplementary material). Respondents were asked to name what they saw, and all their answers were recorded (field-coded and as free text).

147. The ‘desirability’ of the responses was field coded by the survey team. Sample responses (as instructed through the survey manual) for ‘desirable’ answers included, for example, “Only if the doctor says that I should”. Sample responses for ‘undesirable’ answers included “Yes, you can buy it in the shop over there!” The variable should be interpreted as ‘the fraction of respondents who uttered a ‘desirable’ response’—the inverse is the fraction of responses that could not be deemed ‘desirable’ (eg, ‘do not know’ or ‘no opinion’).

148. Because recalled descriptions of medicine tend to be ambiguous, we limited our analysis to medicines where we had a high degree of certainty that they were an antibiotic. This was specifically the case if survey respondents mentioned common antibiotic descriptions such as ‘anti-inflammatory’, ‘amoxi’ or ‘colem’, if they indicated explicitly that they know what ‘anti-inflammatory medicine’ is (noting that the term describes antibiotics unambiguously in Thai), and if they subsequently mentioned any of the previously mentioned antibiotics during their description of an illness episode (conversely, we excluded cases were the medicine could not be confirmed as either antibiotic or non-antibiotic, including descriptions like ‘white powder’ or ‘green capsule’).

149. Aristotle (1954). *Rhetoric*. Translated by Roberts. New York, NY: Modern Library. Original edition, 350 BC.

150. Arya Nielsen et al. (2007). "The effect of gua sha treatment on the microcirculation of surface tissue: a pilot study in healthy subjects." *EXPLORE* no. 3 (5):456-466. doi: 10.1016/j.explore.2007.06.001

151. Nithima Sumpradit et al. (2012). "Antibiotics Smart Use: a workable model for promoting the rational use of medicines in Thailand." *Bulletin of the World Health Organization* no. 90 (12):905-913. doi: 10.2471/BLT.12.105445

152. C Muksong and K. Chuengsatiansup (2020). forthcoming. "Medicine and public health in Thai historiography: from an elitist view to counter-hegemonic discourse." In *Health, pluralism and globalisation: a modern history of medicine in South-East Asia*, edited by Monnais and Cook. London: The Wellcome Trust Centre for the History.

153. L Sringernyuang (2000). Availability and use of medicines in rural Thailand. Amsterdam: Amsterdam Institute for Social Science Research.

154. Although this was not the focus of the current paper, we note for full disclosure that the workshops, too, had mixed behavioural impacts. The poster making sessions in Chiang Rai demonstrated for instance how our conversations about drug resistance and the introduction of messages from the World Health Organization entailed at times problematic interpretations like, “You shouldn’t take medicines that you have never seen before”—the research team responded to such interpretations directly in order to avoid misunderstandings. In addition, previous behavioural analyses documented that, while workshop participants demonstrated higher levels of awareness of drug resistance, alignment of antibiotic use with global health recommendations was mixed, and in one case, a villager started selling antibiotics after the workshop. For more details on the behavioural analysis, see Nutcha Charoenboon et al. (2019) and Marco Haenssgen et al. (2018).

155. For example, Redfern, *et al.*, Spreading the message of antimicrobial resistance: a detailed account of a successful public engagement event.

156. Antoine Boivin et al. (2018). 2018. "Patient and public engagement in research and health system decision making: a systematic review of evaluation tools (epub ahead of print)." *Health Expectations*. doi: 10.1111/hex.12804

157. Staniszewska, *et al.* GRIPP2 reporting checklists: tools to improve reporting of patient and public involvement in research.

158. Jerke, *et al.* Smoking cessation in mental health communities: a living newspaper applied theatre project.

159. Switzer, What’s in an image? Towards a critical and interdisciplinary reading of participatory visual methods.

160. R. C Barfield and L. Selman (2014). "Health and humanities: spirituality and religion." In *Health humanities reader*, edited by Jones, Wear, Friedman and Pachucki, 376-386. New Brunswick, NJ: Rutgers University Press.

161. Abimbola, Beyond positive a priori bias: reframing community engagement in LMICs (epub ahead of print), 1.

162. Marco J Haenssgen et al. (2019)

163. Marc Mendelson et al. (2017). "Antibiotic resistance has a language problem." *Nature* no. 545 (7652):23-25. doi: 10.1038/545023a

164. Haak and Radyowijati, Determinants of antimicrobial use: poorly understood, poorly researched.

165. S Harbarth and D. L. Monnet (2008). "Cultural and socioeconomic determinants of antibiotic use." In *Antibiotic Policies: Fighting Resistance*, edited by Gould and van der Meer, 29-40. Boston, MA: Springer.

166. K Sirijoti, P. Havanond Hongsranagon, and W. Pannoi (2014). "Assessment of knowledge attitudes and practices regarding antibiotic use in Trang province, Thailand." *Journal of Health Research* no. 28 (5):299-307.

167. Ramona K C Finnie et al. (2011). "Factors associated with patient and health care system delay in diagnosis and treatment for TB in sub-Saharan African countries with high burdens of TB and HIV." *Tropical Medicine & International Health* no. 16 (4):394-411. doi: 10.1111/j.1365-3156.2010.02718.x

168. Haak and Radyowijati, Determinants of antimicrobial use: poorly understood, poorly researched.

169. Chandler, Current accounts of antimicrobial resistance: stabilisation, individualisation and antibiotics as infrastructure, 5.

170. S Willson and K. Miller (2014). "Data collection." In *Cognitive interviewing methodology: a sociological approach for survey question evaluation*, edited by Miller, Willson, Chepp and Padilla, 15-34. Hoboken, NJ: Wiley.

171. See Linda Mayoux and Robert Chambers (2005). "Reversing the paradigm: quantification, participatory methods and pro-poor impact assessment." *Journal of International Development* no. 17 (2):271-298. doi: 10.1002/jid.1214

172. Howard S. Becker (1995). "Visual sociology, documentary photography, and photojournalism: It's (almost) all a matter of context." *Visual Sociology* no. 10 (1-2):5-14. doi: 10.1080/14725869508583745

173. J Prosser and D. Schwartz (2005). "Photographs and the sociological research process." In *Image-based research: a sourcebook for qualitative researchers*, edited by Prosser, 101-115. London: Falmer.

174. Treffry-Goatley, *et al.* Community engagement with HIV drug adherence in rural South Africa: a transdisciplinary approach.

175. Switzer, What’s in an image? Towards a critical and interdisciplinary reading of participatory visual methods.

176. Hume, *et al.* Biomedicine and the humanities: growing pains.

177. Jordanova, Medicine and the visual arts, 60.

178. Bleakley, Towards a 'critical medical humanities'.

179. Nutcha Charoenboon et al. (2019)

180. Hume, *et al.* Biomedicine and the humanities: growing pains.

181. J. P Ansloos (2018). “To speak in our own ways about the world, without shame”: reflections on indigenous resurgence in anti-oppressive research.” In *Creating social change through creativity: anti-oppressive arts-based research methodologies*, edited by Capous-Desyllas and Morgaine, 3-18. Cham: Springer.

182. Marco J Haenssgen (2019)

183. Michael Etherton and Tim Prentki (2006). "Drama for change? Prove it! Impact assessment in applied theatre." *Research in Drama Education: The Journal of Applied Theatre and Performance* no. 11 (2):139-155. doi: 10.1080/13569780600670718

184. Susan Galloway (2009). "Theory-based evaluation and the social impact of the arts." *Cultural Trends* no. 18 (2):125-148. doi: 10.1080/09548960902826143

185. Darquise Lafrenière and Susan M Cox (2013). "‘If you can call it a poem’: toward a framework for the assessment of arts-based works." *Qualitative Research* no. 13 (3):318-336. doi: 10.1177/1468794112446104
