# Supplementary material for: Tales of treatment and new perspectives for global health research on antimicrobial resistance
Source: Med Humanit. 2020 Sep 18;47(4):e10. doi: 10.1136/medhum-2020-011894 (PMC8639946; doi:10.1136/medhum-2020-011894)
Supplement: Supplementary data [file medhum-2020-011894supp001.pdf]

OxTREC reference: 528-17 **ANTIBIOTICS AND ACTIVITY SPACES**

1. Village Checklist (GPS coordinates of village and facilities) (to be completed by supervisor)

What kind of facility would you like to record?

A. District Number		[*code entered automatically*]
B. Village Number		[*code entered automatically*]
C. Village centre	a) Latitude	[*coordinates entered automatically*]
	b) Longitude	[*coordinates entered automatically*]
D. Village head's house	a) Latitude	[*coordinates entered automatically*]
	b) Longitude	[*coordinates entered automatically*]
E. Local shop	a) Latitude	[*coordinates entered automatically*]
	b) Longitude	[*coordinates entered automatically*]
F. Market	a) Latitude	[*coordinates entered automatically*]
	b) Longitude	[*coordinates entered automatically*]
G. Temple	a) Latitude	[*coordinates entered automatically*]
	b) Longitude	[*coordinates entered automatically*]
H. School	a) Latitude	[*coordinates entered automatically*]
	b) Longitude	[*coordinates entered automatically*]
I. Bus stop	a) Latitude	[*coordinates entered automatically*]
	b) Longitude	[*coordinates entered automatically*]
J. Health facility<br>Specify (public, private, pharmacy, local store, traditional healer, etc.): _________________	a) Latitude	[*coordinates entered automatically*]
	b) Longitude	[*coordinates entered automatically*]
	c) Who is staffing the facility?	Total staff: ___<br>Staff at time of visit: ________
	d) Does the provider have antibiotics available?	Yes ....1<br>No 0

Haenssgen MJ, *et al. Med Humanit* 2021; 47:e10–e27. doi: 10.1136/medhum-2020-011894

Interview data [*Record observation*]

i. District Number		[*code entered automatically*]
ii. PSU Number		[*code entered automatically*]
iii. Household number		Number: ______
iv. Household coordinates	a) Latitude	[*coordinates entered automatically*]
	b) Longitude	[*coordinates entered automatically*]

v. What type is this house most similar to?

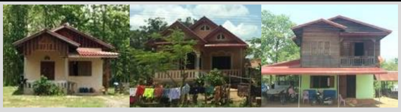

1…

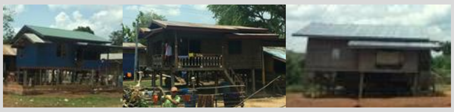

2…

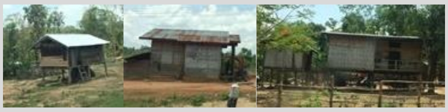

3…

vi. Time of visit	a) First visit	[*time entered automatically*]
	b) Second visit	[*time entered automatically*]

List all persons aged 18+ years in household

Hello, I'm a researcher working for the Mahidol-Oxford Tropical Medicine Research Unit. We are interested in the lives and health behaviours of villagers across Thailand and Lao PDR. We are selecting participants randomly and would like to choose one or two members of your household. In order to choose and ask them to participate, could you please tell us who lives here? [provide PIS on request]

[*1 respondent per every 5 household members will be selected randomly from this list*]

Name	Nickname	Sex (M / F)	Age	Available for interview today? (Yes / No)

Statement of consent (Respondent will receive participant information sheet and verbal consent will be taken)
Thank you for participating. You will receive a small token of gratitude for your participation at the end of the interview.

vii.Date of interview	[*date entered automatically*]
viii.Time of interview begin	[*time entered automatically*]
ix.Respondent name	Respondent name: ____________________________
x.Interviewer code	[*code entered automatically*]

Part I: Personal and Household Characteristics
Let us begin with a few questions about yourself and your household.

**1.** [*record as observed*] Sex		Female........................ 1<br>Male 0
**2.** How old are you? [*in years*] [***If respondent cannot give exact age, ask for approximate age and code in range: 18-24, 25-34, 35-44, 45-59, 60 and older***]		Age in years: _______
**3.** Please indicate what kind of work you do. If you have more than one occupation at one time or throughout the year, please begin with the one in which you spend the most time and name up to three. If you do not have an occupation, please also mention whether you are still a student, retired, or unemployed.	a) Main occupation	Occupation: _________
	b) Side occupation	Occupation: _________
	c) Side occupation	Occupation: _________
**4.** What is your mother tongue?		Mother tongue: ________________
**5.** [In Thailand:] Can you speak Thai? [In Laos:] Can you speak Lao?		Yes .... 1<br>No 0
**6.** What is the highest grade of schooling that you completed?<br>[***excluding informal education and pre-school education such as nursery and kindergarten, but including grade school, high school, vocational training, tertiary education, etc.***]		Highest grade: ___
**7.** Are you the head of your household?		Yes ....1<br>No 0
**7.1.** [*if no*] What is the name of your household head?		Name: __________

**8.** What is your current marital status?	Never married........ 1<br>Currently married........ 2<br>Cohabiting........ 3<br>Separated / divorced........ 4<br>Widowed........ 5	
**9.** Are there any close family members of yours [children, spouse, siblings, parents] who live elsewhere? [*select "no" if not applicable*]	**9.1.** Do your parents live outside of this village? [*do not count parents-in-law*]	At least 1 person outside village .... 1<br>All inside village / not applicable.... 0
	**9.2.** Does your spouse live outside of this village?	At least 1 person outside village .... 1<br>All inside village / not applicable.... 0
	**9.3.** Do you have siblings who live outside of this village? [*do not count brothers-in-law and sisters-in-law*]	At least 1 person outside village .... 1<br>All inside village / not applicable.... 0
	**9.4.** Do you have children who live outside of this village?	At least 1 person outside village .... 1<br>All inside village / not applicable.... 0
Part II: Social Networks [for network census villages only]<br>I will now ask you some questions about your interactions with other people within and outside of your village.		
**10.** [Round I of network survey only] Where do you spend most of your time interacting with other people from your village?	a) Field: ___<br>b) Temple: ___<br>c) Local store: ___<br>d) Market: ___<br>e) Children's schools: ___<br>f) Home: ____<br>g) Workplace: ___<br>h) Village event/s: ______<br>i) Other site: ___	

11. **[Round I of network survey only]** Outside your household, with whom do you interact on a regular basis? (May be anyone from both inside and outside of the village, and through any platform which might not require a face-to-face interaction)

	a) What is the nickname of the person?	b) How is this person related to you? [*give examples if respondent is unsure about answer categories*]	c) What is the sex of this person?	d) Where does this person live?	e) What is the name of the household head of this person?	f) How often do you interact with this person?	g) How do you interact with this person? [*Mark all that apply*]	h) Do your conversations relate to health and well-being?
11.1. Contact 1	Nickname _____<br>Name _____	Spouse............ 1<br>Parent............ 2<br>Child............ 3<br>Sibling............ 4<br>Other relative............ 5<br>Neighbour............ 6<br>Friend (if not neighbour)............ 7<br>Other villager............ 8<br>Other (specify) _............ 9	Female...1<br>Male 0	In village .... 1<br>(specify: _____)<br>Outside village .. 2	Name of household head<br>________	Daily or more often ....4<br>Weekly or few times/week ....3<br>Monthly or few times/month ...2<br>Yearly or few times/year ....1<br>Less often or never 0	Face-to-face...1<br>Voice call........2<br>Messenger .....3<br>Other (specify)<br>_____ 4	Yes .... 1<br>No 0
11.2. Contact n	Nickname<br>Name	1 2 3 4 5 6 7 8 9	1 0	1 2	Name	0 1 2 3 4	1 2 3 4	1 0

**11a. [Round II of network survey only]** When we last visited you, you told us that you interact regularly with [*names*]. Has anything changed since last time?	Yes ....1 → **[update social network question 11]**<br>No..........0
**11i. [Round I of network survey only]** Is there anybody in your household with whom you talk about health and well-being? [***Mark all that apply***]	[***mark all names from household roster that apply***]

[*For network survey village respondents in Round 2*]<br>12. An education activity has recently taken place in your village.	
12.1. Did you participate in any of the activities?	Yes .... 1<br>Yes, but not throughout .... 2<br>No .... 3<br>Don't know / prefer not to say 4
12.2. Did you talk with anybody about the activity in your village?<br>[*"Talking" can involve any conversation including asking for information, informing about the educational activity, or discussing it (regardless of actual attendance)*]	a) Nickname 1: ______ b) Full name 1: ______ c) Relationship 1: 1 2 3 4 5 6 7<br>a) Nickname n: ______ b) Full name n: ______ c) Relationship n: 1 2 3 4 5 6 7<br>[*Relationship codes*]<br>Household member .... 1<br>Family member outside HH .... 2<br>Other relative .... 3<br>Neighbour .... 4<br>Friend other than neighbour .... 5<br>Other villager .... 6<br>Other (specify) _ 7
[*If respondent indicates conversation in Q 12.2*]<br>12.3. What subjects did you talk about in respect to the activity?<br>[*mark all that apply*]	Going to doctor when sick .... 1<br>Anti-inflammatories/antibiotics .... 2<br>Germs .... 3<br>Using medicines correctly .... 4<br>Activity in general .... 5<br>Games/awards .... 6<br>Song/Story/Play .... 7<br>Money/compensation .... 8<br>Other (specify) ____ 9

Part III: Healthcare Seeking Thank you for this. Now we come to a part where I will ask you some questions about health and health providers around here.

13. I would now like to ask you about the sources of health advice and medicine or other treatment that are available to you. Please think about all the places where you can go to get advice, treatment, or drugs if you (or your children) are sick.<br><br>Do you consider the following providers when you (or your children) feel unwell?<br><br>[*Mark all that apply*]	13.1. Drug dispensary, other local store selling medicine	Consultation .... 1<br>Medical advice .... 2<br>Access to medicine .... 3<br>Other reason(s) .... 4<br>Don't consider this provider .... 98<br>Don't know such a provider 99
	13.2. Traditional healer	Consultation .... 1<br>Medical advice .... 2<br>Access to medicine .... 3<br>Other reason(s) .... 4<br>Don't consider this provider .... 98<br>Don't know such a provider 99
	13.3. Pharmacist	Consultation .... 1<br>Medical advice .... 2<br>Access to medicine .... 3<br>Other reason(s) .... 4<br>Don't consider this provider .... 98<br>Don't know such a provider 99
	13.4. Private clinic	Consultation .... 1<br>Medical advice .... 2<br>Access to medicine .... 3<br>Other reason(s) .... 4<br>Don't consider this provider .... 98<br>Don't know such a provider 99
	13.5. Private hospital	Consultation .... 1<br>Medical advice .... 2<br>Access to medicine .... 3<br>Other reason(s) .... 4<br>Don't consider this provider .... 98<br>Don't know such a provider 99
	13.6. Health volunteer	Consultation .... 1<br>Medical advice .... 2<br>Access to medicine .... 3<br>Other reason(s) .... 4<br>Don't consider this provider .... 98<br>Don't know such a provider 99
	13.7. Public primary care unit	Consultation .... 1<br>Medical advice .... 2<br>Access to medicine .... 3<br>Other reason(s) .... 4<br>Don't consider this provider .... 98<br>Don't know such a provider 99

Haenssgen MJ, *et al. Med Humanit* 2021; 47:e10–e27. doi: 10.1136/medhum-2020-011894

	**13.8.** Public hospital	Consultation .... 1<br>Medical advice........ 2<br>Access to medicine........ 3<br>Other reason(s) .... 4<br>Don't consider this provider........ 98<br>Don't know such a provider 99
	**13.9.** Other providers or Internet? Specify: ________	Consultation .... 1<br>Medical advice........ 2<br>Access to medicine........ 3<br>Other reason(s) .... 4<br>Don't consider this provider........ 98<br>Don't know such a provider 99

14. Now if you think again, is there anyone else with whom you talk about health?

	a) What is the nickname of the person?	b) What is the full name of the person?	c) How is this person related to you? [*give examples if respondent is unsure about answer categories*]	d) What is the sex of this person?	e) Where does this person live?	f) What is the name of the household head of this person?	g) How often do you interact with this person?	h) How do you interact with this person? [*Mark all thatapply*]
**14.1.** Contact 1	Name ______	Name ______	Spouse.......... 1<br>Parent .... 2<br>Child .... 3<br>Sibling.......... 4<br>Other relative .... 5<br>Neighbour.......... 6<br>Friend (if not neighbour).......... 7<br>Other villager.......... 8<br>Other (specify) _.......... 9	Female ..1<br>Male 0	In village .... 1 (specify: _____)<br>Outside village .. 2	Name of household head ________	Daily or more often ....4<br>Weekly or few times/week ....3<br>Monthly or few times/month ...2<br>Yearly or few times/year ....1<br>Less often or never..........0	Face-to-face.......... 1<br>Voice call.......... 2<br>Messenger .... 3<br>Other (specify) _____ 4
**14.2.** Contact n	Name	Name	1 2 3 4 5 6 7 8 9	1 0	1 2	Name	0 1 2 3 4	1 2 3 4

Haenssgen MJ, *et al. Med Humanit* 2021; 47:e10–e27. doi: 10.1136/medhum-2020-011894

**15.** Did you or a child in your household have an acute illness (not a chronic, long-term condition that comes again and again) or an accident in the last two months? If yes, I will ask you about these illnesses one-by-one. [***if no, continue with Question 19***]	No........................0 ➔ [*Q 16*]<br>Yes......................1 ↓
[***if yes***:]<br>**15.a** [Confirm if this episode is for respondent or child]	Respondent ....1 ➔ [***Q 15.1***]<br>Child 2
**15.b** How old is the child?	Age in years: _______
**15.c** Is the child female or male	Female ....1<br>Male 0
**15.1.** Can you please describe the symptoms or problem in your own words?	Description of condition:<br>____
**15.2.** Did [you / the child] receive a diagnosis of the illness from any medical provide, friend, or internet source?<br><br>If so, can you please describe the diagnosis of the illness if you received any and where [you / the child] received it? [***note: the diagnosis might be given by any medical provider including untrained and informal. Record all diagnoses if more than one.***]	a) Diagnosis 1: ___________ b) Medical provider 1: 1 2 3 4 5 6 7 8<br>a) Diagnosis n: ___________ b) Medical provider n: 1 2 3 4 5 6 7 8<br><br>[***Response codes***]<br>Drug dispensary, other local store selling medicine ....1<br>Traditional healer........................2<br>Pharmacist ....3<br>Private clinic........................4<br>Private hospital ....5<br>Primary care unit ....6<br>Public hospital........................7<br>Other providers or Internet? Specify: ________........................8
**15.3.** When did [you / the child] experience the accident/discomfort (for the first time)	Onset: ___ days / ______ weeks / _____ months ago
**15.4.** Would you describe the illness/accident as "mild," "moderate," or "severe"?	Mild..1<br>Moderate...2<br>Severe 3

15.5. Can you please explain the stages of the treatment? I will ask you step-by-step what you did, starting from the moment [you / the child] first experienced a discomfort.

	**15.5.1.** Step 1 (detection)	Step n
a) What kind of help or treatment did you get at this stage?<br>[***if unsure, specify***]	Ignored /did nothing ....1<br>Self-care (sleep, rest, medicine at home) ....2<br>Care from family and friends (full-time)........................3<br>Treated/consulted at a traditional healer ....4<br>Treated/cons. at a pharmacist........................5<br>Treated/cons. at shop selling drugs........................6<br>Treated/cons. at priv. clinic/hospital ....7<br>Treated/cons. at primary care unit........................8<br>Treated/cons. at a gvt. Hospital........................9<br>Other (specify) _____ 10	1<br>2<br>3<br>4<br>5<br>6<br>7<br>8<br>9<br>10
b) Where did this activity take place?	At home ....1<br>Less than 10 min. from home........................2<br>10 to 29 min. ....3<br>30 to 59 min. ....4<br>60 to 119 min. ....5<br>2 hours or more from home........................6	1<br>2<br>3<br>4<br>5<br>6
c) How did [you / the child] get to the place of the activity? [***select "at home" according to prior responses***]	At home .... 1<br>Walk .... 2<br>Own bicycle .... 3<br>Own motorcycle / Three-wheeler........................ 4<br>Own car / four-wheeler .... 5<br>Taxi or other hired ride........................ 6<br>Public transport........................ 7<br>Other (specify) ___ 8	1<br>2<br>3<br>4<br>5<br>6<br>7<br>8
d) How long did this stage last?<br>[***let respondent choose category; if <1 day, code "1" day***]	Duration:<br>___ days<br>___ weeks<br>___ months	___ days<br>___ weeks<br>__ months
e) Can you please name or describe all the medicines that you received or were prescribed during this step?<br><br>[***include medicine stored at home if "self-care at home"***] [***continue for all medicines received, then complete Questions g to k for each medicine individually***] 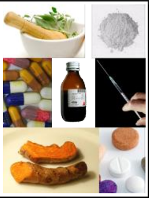	Medicine 1:<br>Name/description: _____<br><br>Medicine n:<br>Name/description: _____	Medicine 1<br><br>Medicine n
f) For how long did [you / the child] take the medicine?<br>[***let respondent choose category; if more than one repeated episode, indicate total duration***]<br>[***for each medicine individually***]	Duration:<br>___ days<br>___ weeks<br>___ months	___ days<br>___ weeks<br>__ months

g) How often per day did [you / the child] take the medicine?<br><br>[*calculate into daily use according to respondent's chosen frequency*]<br>[*for each medicine individually*]	Frequency: ___ times daily	___ times daily
h) What dosage did [you / the child] normally take?<br>[*let respondent choose category according to type of medicine*]<br>[*for each medicine individually*]	Dosage<br>___ tablets / capsules<br>___ drops (for liquid medicine)<br>___ spoons (for liquid medicine)<br>____ shots/injections (for intravenous medicine)<br>per time administered	___ tablets<br>___ drops<br>___ spoons<br>___ shots
i) Did [you / the child] take the medicine exactly as it was recommended to you by the person who prescribed/sold them<br>[*for each medicine individually*]	Yes .... 1<br>No .... 0<br>Did not receive advice .... 9<br>Don't know 99	1<br>2<br>9<br>99
j) Did [you / the child] finish the medicine?<br>[*for each medicine individually*]	Yes .... 1<br>No 0	1<br>0
k) Did you or anybody else use a mobile phone during this stage <u>in connection with your condition</u>? [*if no, go to next step*]	Yes .... 1<br>No .... 0 ➔ [*next step*]	1<br>0
l) What was the purpose of using the mobile phone?<br><br>[*Mark all that apply*]	Ask for advice .... 1<br>Call for treatment .... 2<br>Arrange transport .... 3<br>Appointment .... 4<br>Reassure family/friends .... 5<br>Ask for money/supplies .... 6<br>Provider contacting me for information .... 7<br>Treatment reminder .... 8<br>Other (specify) _ 9	1<br>2<br>3<br>4<br>5<br>6<br>7<br>8<br>9
m) Which mobile phone functions did you or anybody else use?<br>[*Mark all that apply*]	Call .... 1<br>SMS .... 2<br>Internet, messenger .... 3<br>Alarm, calendar, reminder, etc. .... 4<br>Other (specify) _ 5	1<br>2<br>3<br>4<br>5

**15.6.** [Have you / has the child] now recovered from the illness/accident?	Yes .... 1<br>No 0
**15.7.** Was anybody of your personal relationships involved in providing advice or help <u>during the illness</u>? [*record up to ten names*]	Yes .... 1<br>No 0
[*For district survey*]<br>**15.7.b** How are these people related to you? [*Mark all that apply*]	Spouse .... 1<br>Parent .... 2<br>Child .... 3<br>Sibling .... 4<br>Other relative .... 5<br>Neighbour .... 6<br>Friend (if not neighbour) .... 7<br>Other villager .... 8<br>Other (specify) _ 9
**15.7.c** What kind of support did they provide? [*Mark all that apply*]	Providing healthcare/attending .... 11<br>Providing advice .... 12<br>Providing medicine .... 13<br>Lending/granting money .... 21<br>Transportation/Lending vehicle .... 22<br>Contacting family/friends .... 23<br>Providing food .... 31<br>Helping with children/housework .... 32<br>Helping with jobs/agriculture work (feeding animals/tending crops/covering shifts, etc.) 33<br>Other (specify) _ 99

[*For network survey*]	a) What is the name of the person?	b) How is this person related to you?	c) What kind of support was provided?<br>[*mark all that apply*]
**15.7.1.**<br>Contact 1	Name:<br>_______	Spouse .... 1<br>Parent .... 2<br>Child .... 3<br>Sibling .... 4<br>Other relative .... 5<br>Neighbour .... 6<br>Friend (if not neighbour) .... 7<br>Other villager .... 8<br>Other (specify) _ 9	Providing healthcare/attending .... 11<br>Providing advice .... 12<br>Providing medicine .... 13<br>Lending/granting money .... 21<br>Transportation/Lending vehicle .... 22<br>Contacting family/friends .... 23<br>Providing food .... 31<br>Helping with children/housework .... 32<br>Helping with jobs/agriculture work (feeding animals/tending crops/covering shifts, etc.) 33<br>Other (specify) _ 99
**15.7.2.**<br>Contact n	Name	1 2 3 4 5 6 7 8 9	11 12 13 21 22 23 31 32 33 99

Haenssgen MJ, *et al. Med Humanit* 2021; 47:e10–e27. doi: 10.1136/medhum-2020-011894

**15.8.** Did <u>you</u> have another acute illness (not a chronic, long-term condition that comes again and again) or an accident <u>in the last two</u> months?<br>[***if yes, complete another sheet for Question 15***]	Yes .... 1 ➔ [*Q 15*]<br>No .... 0 🡻
**16.** I would now like to ask you your opinion about medicine. There are no right or wrong answers, I only want to understand what you think. Consider the following medicines:	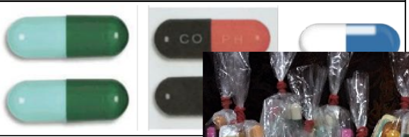 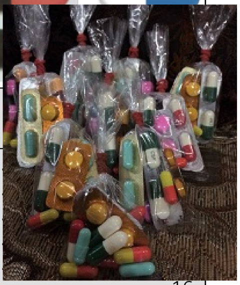
**16.1.** Have you seen these medicines before?	Yes .... 1<br>No .... 0 ➔ [*Q 16.4*]
**16.2.** What do you call this medicine?	Antibiotics ท่านเรียกยานี้ว่าอะไร........ [illegible]<br>Anti-inflammatory ยาแก้อักเสบ........ [illegible]<br>Germ killer ยาฆ่าเชื้อ .... [illegible]<br>Amoxy / Amoxicillin อะม็อกซี่/อะม็อกซี่ซิลลิน .... [illegible]<br>Sore throat medicine ยาแก้เจ็บคอ .... [illegible]<br>Cough medicine ยาแก้ไอ .... 16<br>Pain reliever ยาแก้ปวด .... 17<br>Fever reliever ยาแก้ไข้ .... 18<br>Other (specify: _____) อื่นๆ (โปรดระบุ) .... 98<br>Germ preventer / antibiotic ຢາຕ້ານເຊື້ອ........ 21<br>Amok ຢາຕ້ານເຊື້ອ .... 22<br>Ampi ຢາແອມປີ .... 23<br>Tetra ຢາເຕຕ້າ .... 24<br>Gulolam ກູໂລລາມ........ 25<br>Sepasin ເຊພາຊິນ........ 26<br>Other (specify: __)........ 99
**16.3.** What symptoms or illnesses would you use this medicine for?	Fever .... 1<br>Cough .... 2<br>Sore throat........ 3<br>Inflammation .... 4<br>Cold, flu, runny nose .... 5<br>Diarrhoea .... 6<br>Headache .... 7<br>Stomach ache .... 8<br>Muscle pain, other aches........ 9<br>Skin diseases, rashes, lumps .... 10<br>Wounds........ 11<br>Urinary tract infections .... 12<br>Every kind of sickness........ 13<br>Whatever the doctor suggests .... 14<br>Don't know / prefer not to say .... 98<br>Other (specify: __)........ 99
**16.4.** Is there any situation for which you would buy this medicine?	Desirable attitude/knowledge .... 1<br>Undesirable attitude/knowledge .... 0<br>No attitude / refuse to answer (respondent is aware, but doesn't reveal attitude)........ 97<br>Answer does not apply to question (respondent may be aware/unaware; satisficing)...... 98<br>Not aware of this medicine (awkward, cannot answer but does not try to satisfy) 99
**16.5.** Do you prefer other remedies such as herbs or cough syrup to this medicine for [sore throat]?	Desirable attitude/knowledge .... 1<br>Undesirable attitude/knowledge .... 0<br>No attitude / refuse to answer (respondent is aware, but doesn't reveal attitude)........ 97<br>Answer does not apply to question (respondent may be aware/unaware; satisficing)...... 98<br>Not aware of this medicine (awkward, cannot answer but does not try to satisfy) 99
**16.6.** If you were prescribed this medicine by a doctor and did not finish the course, would you keep it for future use?	Desirable attitude/knowledge .... 1<br>Undesirable attitude/knowledge .... 0<br>No attitude / refuse to answer (respondent is aware, but doesn't reveal attitude)........ 97<br>Answer does not apply to question (respondent may be aware/unaware; satisficing)...... 98<br>Not aware of this medicine (awkward, cannot answer but does not try to satisfy) 99
**16.7.** Have you heard about drug resistance?<br>(**16.7a** using alternative term "lueng yah" in Lao)	Yes .... 1<br>No 2
**16.8.** What do you think is drug resistance?<br>(**16.8a** using alternative term "lueng yah" in Lao)	Bacteria are resistant to medicine .... 1<br>Antibiotics become less effective if used wrongly/too much........ 2<br>Medicine in general becomes less effective if used wrongly/too much........ 3<br>Being stubborn to take medicine .... 4<br>Being addicted to medicine........ 5<br>Drug allergy .... 6<br>Lueng yah (drug resistance) .... 7<br>Answer does not relate to drug resistance........ 8<br>Other (specify)........ 98<br>"Don't know"........ 99
**16.9.** Can your drug resistance ("*due yah*") spread to other people, for example if you sneeze on them?	Desirable attitude/knowledge .... 1<br>Undesirable attitude/knowledge .... 0<br>No attitude / refuse to answer (respondent is aware, but doesn't reveal attitude)........ 97<br>Answer does not apply to question (respondent may be aware/unaware; satisficing)...... 98<br>Not aware of this medicine (awkward, cannot answer but does not try to satisfy) 99

Part IV: Household assets
We now come to the last part. Can you please provide me with some information about your household?

**17.** How many rooms does this house have apart from toilet and hallways?	Number of rooms: ________
**18.** What is the electricity situation in your household on a typical day?	Power at all times, no power cuts (90-100%) .... 1<br>Power most of the time, occasional power cuts (>50%) .... 2<br>Power sometimes, frequent power cuts (<50%) .... 3<br>No electricity 4
**19.** What kind of toilet does this house have and is it shared with other people in this community? *[if more than one, choose "best" toilet] [use show card to facilitate answers]*	Unshared flush toilet (e.g. piped sewer system, septic tank, pour flush toilet)........ 1<br>Shared (flush or non-flush) toilet with other community members or public toilet 2<br>No facility, Bush, Field, or others 3
**20.** What is the drinking water source of this house and is it shared with other people in this community? [*use show card to facilitate answers*]	Water piped into house or yard.......... 1<br>Water not directly piped into house or yard (e.g. well, borehole, water from spring, rainwater, tanker truck, surface water including rivers, bottled water, etc.)2
**21.** What kind of fuel does this household use for cooking?	Improved fuel source (e.g. Electricity, gas stove, etc.) .... 1<br>Unimproved fuel source (e.g. Coal / Lignite, Charcoal, Wood, Straw / Shrubs / Grass, Animal dung, Agricultural crop residue) .... 2<br>No food cooked in household 3

**22.** I will now ask you for some items in your household. Please tell me…	Number of items in household:	
	**22.1.** Have you got a *functioning* **radio** in your household? If so, how many?	__
	**22.2.** Have you got a *functioning* **TV** in your household? If so, how many?	__
	**22.3.** Have you got a *functioning* **rice cooker** in your household? If so, how many?	__
	**22.4.** Have you got a *functioning* **landline telephone** in your household? If so, how many?	__
	**22.5.** Have you got a *functioning* **mobile phone** in your household? If so, how many?	__
	**22.6.** Have you got a *functioning* **computer** in your household? If so, how many?	__
	**22.7.** Have you got a *functioning* **bicycle** in your household? If so, how many?	__
	**22.8.** Have you got a *functioning* **scooter, motorcycle, or tricycle** in your household? If so, how many?	__
	**22.9.** Have you got a *functioning* **car or truck** in your household? If so, how many?	__
	**22.10.** Have you got a *functioning* **tractor** in your household? If so, how many?	__
	**22.11.** Have you got a *functioning* **refrigerator or freezer** in your household? If so, how many?	__

**23.** How long does it normally take you to get to the following places?	**23.1.** How long does it take to get to the nearest market?	Less than 10 minutes .... 1<br>10 to 29 minutes .... 2<br>30 to 59 minutes .... 3<br>60 to 119 minutes .... 4<br>2 hours or more.......... 5
	**23.2.** How long does it take to get to the village hall or the village head's house?	Less than 10 minutes .... 1<br>10 to 29 minutes .... 2<br>30 to 59 minutes .... 3<br>60 to 119 minutes .... 4<br>2 hours or more.......... 5
	**23.3.** How long does it take to get to the nearest public or private doctor?	Less than 10 minutes .... 1<br>10 to 29 minutes .... 2<br>30 to 59 minutes .... 3<br>60 to 119 minutes .... 4<br>2 hours or more.......... 5

**24.** What is your religion?	No religion ....0<br>Buddhist..........1<br>Christian..........2<br>Muslim ....3<br>Spirit (religious belief in Lao) ....4<br>Other (Specify) __________..........5<br>Don't know 99
**25.** What is your nationality?	Thai ....1<br>Lao..........2<br>Myanmar/Burmese..........3<br>Chinese ....4<br>Other (Specify) __________..........9<br>Don't know 99

**26.** What is your ethnic background?	Thai ....1<br>Tai Yai ....2<br>Akha (E-Koh) ....3<br>Pakakeryor (Karen) ....4<br>Lahu (Muser) ....5<br>Lisu (Lisaw) ....6<br>Hmong (Meaw) ....7<br>Mien (Yao) ....8<br>Burmese ....9<br>Yunnan (Jin Haw) ....10<br>Tai Lue (Tai) ....11<br>Lao ....21<br>Kathuic ....22<br>Bahnaric Khmer ....23<br>Tai Thai ....24<br>Other (Specify) ________ ....30<br>Don't know 99
xi. Interview end time	[*time entered automatically*]
Thank you very much for participating in this survey. [***give gift to respondent***]	
Part V: Interviewer observations [to be completed by interviewer after interview]	
xii. Was the interview completed?	Yes ....1<br>Yes, with difficulties ....2<br>No 3
xiii. Was someone else present during the interview? [*mark all that apply*]	Survey supervisor ....1<br>Other household or family member ....2<br>Medical practitioner ....3<br>Government officer ....4<br>Other (specify) ____ ....5<br>No one 0
xiv. What is your evaluation of the accuracy and trustworthiness of the informant's answers?	Very good ....1<br>Satisfactory ....2<br>Doubtful ....3<br>Very low 4
xv. Were there any unusual circumstances during the interview?	Please describe: ____________________________________

Haenssgen MJ, *et al. Med Humanit* 2021; 47:e10–e27. doi: 10.1136/medhum-2020-011894
