# Supplementary material for: Tales of treatment and new perspectives for global health research on antimicrobial resistance
Source: Med Humanit. 2020 Sep 18;47(4):e10. doi: 10.1136/medhum-2020-011894 (PMC8639946; doi:10.1136/medhum-2020-011894)
Supplement: Supplementary data [file medhum-2020-011894supp002.pdf]

APPENDIX

Table A1. Comparison of antibiotic sources and use during acute illnesses and accidents across five age groups.

	Chiang Rai Village (1st survey round)						All Three Workshop Villages						Rural Chiang Rai Province					
	18-24	25-34	35-44	45-59	60+	*p*-Value	18-24	25-34	35-44	45-59	60+	*p*-Value	18-24	25-34	35-44	45-59	60+	*p*-Value
**All illness episodes**																		
Number	9	17	33	28	28		65	119	143	215	155		32	53	82	210	194	
% received antibiotics	11.1%	23.5%	15.2%	17.9%	7.1%	0.798	15.4%	20.2%	13.3%	13.0%	12.3%	0.086	5.4%	16.7%	30.1%	20.1%	14.8%	0.059
**All antibiotic use episodes**																		
Number	1	4	5	5	2		10	24	19	28	19		3	11	25	53	33	
% of antibiotic use episodes received from formal sources	100.0%	50.0%	100.0%	60.0%	100.0%	0.291	60.0%	70.8%	84.2%	75.0%	78.9%	0.511	100.0%	42.4%	87.3%	96.2%	84.5%	0.001
% of antibiotic use episodes received from informal sources	0.0%	50.0%	0.0%	40.0%	0.0%	0.291	20.0%	37.5%	15.8%	28.6%	21.1%	0.532	0.0%	14.2%	2.5%	3.8%	1.6%	0.418
% of illness episodes with at least one instance of unfinished antibiotics	100.0%	75.0%	0.0%	60.0%	50.0%	0.102	70.0%	41.7%	21.1%	32.1%	52.6%	0.020	46.9%	13.1%	31.8%	53.4%	26.4%	0.110
% of episodes with at least one instance of strict adherence to antibiotic instructions	100.0%	75.0%	80.0%	20.0%	100.0%	0.240	50.0%	70.8%	78.9%	60.7%	68.4%	0.107	40.3%	31.3%	87.5%	74.8%	81.8%	0.006

Source: Authors, derived from survey data.
Notes. Data on illness episode level. Multiple illness episodes per respondent possible. Provincial-level results are population weighted using census data. *p*-values calculated using Pearson X^2 test
